# A Viral Long Non-Coding RNA Modulates Viral RNA Silencing Suppressor and DCL4-Associated DRB4 Protein Interaction

**DOI:** 10.3390/v18070801

**Published:** 2026-07-20

**Authors:** Amélie Janzam, Lucie Bellott, Johana Chicher, Philippe Hammann, Camille Kempff, Line Jambois, Kamal Hleibieh, Fabrice Michel, Véronique Ziegler-Graff, David Gilmer

**Affiliations:** 1 Institut de Biologie Moléculaire des Plantes, CNRS UPR2357, Université de Strasbourg, 67000 Strasbourg, France; amelie.jk10@gmail.com (A.J.); lucie.bellott@gmail.com (L.B.); camille.kempff123@gmail.com (C.K.); linejambois@gmail.com (L.J.); fabrice.michel@ibmp-cnrs.unistra.fr (F.M.); veronique.ziegler-graff@ibmp-cnrs.unistra.fr (V.Z.-G.); 2 Institut de Biologie Moléculaire et Cellulaire, CNRS, Université de Strasbourg, 67000 Strasbourg, France; j.chicher@ibmc-cnrs.unistra.fr (J.C.); p.hammann@ibmc-cnrs.unistra.fr (P.H.)

**Keywords:** RNAi, VSR, BNYVV, rhizomania, transitivity

## Abstract

The expression of the BNYVV silencing suppressor (VSR) p14 protein, together with the production of a viral non-coding RNA (ncRNA3), affects the long-distance movement on natural host Beta species. Through immunoprecipitation of Flag-p14 and expression of TurboID-p14 coupled to mass spectrometry, we identified several potential p14 cellular partners. These include proteins related to RNA metabolism, proteasome activation, and notably, SGS3—a key player in the siRNA transitivity pathway—and DRB4, the DCL4 cofactor described in *A. thaliana* silencing pathways. The interaction between p14 and DRB4 was specifically retrieved with the hypomorphic p14BA2 VSR mutant in nucleoli and was disrupted in the presence of ncRNA3, a condition that allows the viral mutant to move long distances. Subcellular fractionation of infected tissues revealed that ncRNA3 accumulates in the nucleus through its interaction with p14, while genomic RNA3 remains cytoplasmic. Our results suggest that a specific viral RNA is targeted to the nucleus to participate in silencing suppression function by destabilizing the p14-DRB4 complex. Moreover, the interaction between p14 and SGS3 provides a mechanistic explanation of the role of this VSR in silencing transitivity.

## 1. Introduction

RNA interference (RNAi) controls development, stress responses and genomic integrity while also being responsible for antiviral defence, particularly in plants. Among the actors involved in RNAi, the Dicer-like proteins (DCL) are essential for small RNA biogenesis such as microRNAs (miRNAs) and small interfering RNAs (siRNAs). *Arabidopsis thaliana* encodes four DCL proteins that regulate both transcriptional and post-transcriptional gene expression. DCL1 is mainly involved in miRNA synthesis [1], DCL3 is responsible for the production of endogenous 24 nts long siRNAs [2], and DCL4 generates transacting-siRNA (tasiRNA) [3]. In the context of classical antiviral defences, ensured by DCL4 and DCL2 [4], one strand of viral siRNA duplexes (vsiRNAs) loaded into an Argonaute (AGO) protein targets a complementary sequence. In most cases, target viral RNAs are cleaved by AGO slicing activity. In plants, cleavage products serve as templates for RNA-dependent RNA polymerase (RDR) activity, mainly mediated by RDR6, associated with its cofactor SGS3 (suppressor of gene silencing 3) to ensure de novo double-stranded RNA (dsRNA) synthesis, further cleaved by DCL2 and DCL4 to generate secondary vsiRNAs. This amplification mechanism of the RNA silencing signal, known as transitivity, enhances the efficiency and systemic spread of RNA silencing. The dicing of dsRNA by DCL proteins requires the formation of complexes with their cognate dsRNA binding proteins (DRBs). Seven DRBs are present in *A. thaliana*. Among them, DRB1-DCL1 is certainly the best characterized complex. DRB1 is mandatory for an accurate processing of primary miRNAs [5] and participates in RNA-induced silencing complex (RISC) formation [6]. While DRB2, DRB3 and DRB5 are implicated in non-canonical miRNA biogenesis pathways [7], DRB4 is essential for DCL4 dicing activity in vitro and in vivo [3,8] and is crucial for the plant antiviral defence pathway [9,10,11].

In response to RNA silencing activation, viruses strike back by producing viral suppressors (VSRs) that inhibit various stages of this defence mechanism. *Beet necrotic yellow vein virus* (BNYVV) belonging to the *Benyviridae* family is a multipartite virus possessing up to five positive-sense single-stranded RNAs. Among the housekeeping genes encoded by RNA1 and 2 [12], the VSR p14 acts on the transitivity pathway [13]. RNA3 is mandatory to fulfil a systemic infection on *Beta* host species [14,15,16] thanks to the Coremin motif involved in exoribonuclease XRN4 stalling and subsequent non-coding RNA3 (ncRNA3) accumulation. Hence, the p14 VSR, Coremin motif and ncRNA3 are required for *Beta* species systemic infection [15,17]. The systemic movement of a viral hypomorphic mutant *p14BA2* carrying K^78^A-K^79^A substitution within the zinc-finger domain is only possible on *N. benthamiana* RdR6i plants or in the presence of RNA3 able to produce the ncRNA3 and not in the presence of RNA3E carrying a mutation preventing ncRNA accumulation. This is of particular interest as it indicates that transitivity restricts viral infection in accordance with the reduction in secondary siRNA accumulation by p14 VSR [13,16].

To further study BNYVV VSR function, we deployed immunoprecipitation and TurboID approaches coupled to mass spectrometry to identify p14 potential cellular partners. In particular, we identified DRB4 and SGS3 proteins, both involved in plant antiviral defence. We showed that DRB4 overexpression affected p14 protein subcellular localization and found that a stable DRB4-*p14BA2* interaction was sensitive to ncRNA3 accumulation. Finally, the SGS3 and p14 interaction was investigated.

## 2. Materials and Methods

### 2.1. Plant Growth Conditions

*N. benthamiana* plants were grown in a controlled growth chamber with 16 h light and 8 h dark photoperiod at 22 °C. *N. benthamiana* wt and RdR6i were a gift from Sir David Baulcombe (University of Cambridge, Cambridge, UK).

### 2.2. Isolated Genes

DRB4 (accession number: Niben101Scf06376g01010), fibrillarin (accession number: AM269909) and SGS3 genes (accession number: gb|KJ190939.1) were isolated by RT-PCR with oligo(dT) from healthy *N. benthamiana* using PCR primers ForDRB4 and RevDRB4; ForFib and RevFib; SGS3-gwF and SGS3-gwR (Supplementary Table S1).

### 2.3. Plasmids, Cloning Strategies and In Vitro Transcription

Linearized plasmids containing cDNA copies of RNA1, RNA2 (wild-type (WT) or expressing hypomorphic mutants *p14BA2* or *p14BA3*), RNA3 and RNA3E were used for in vitro transcription as described previously [18,19]. Agroinfectious clones of BNYVV RNA3 and RNA3E were obtained previously in the pJL89 backbone [20]. Transient expression of proteins through agroinfiltration were performed using the pEAQ-Δp19 backbone vector developed for Golden Gate assembly or the pJL89 vector for the TurboID experiment obtained by Gibson assembly. The coding sequences of NbDRB4 and AtDRB4 fused to tRFP were cloned into a Golden Gate-compatible pEAQ vector derived from the pEAQ expression system described by Sainsbury et al. [21]. The expression cassettes were placed under the control of the Cauliflower mosaic virus (CaMV) 35S promoter, and the binary vector conferred kanamycin resistance. For yeast two-hybrid, p14 and hypomorphic mutants were obtained from Chiba et al. 2013 [13], and NbDRB4 was fused to HA tag in pGBK-T7 plasmid using *Eco*RI and *Sal*I restriction sites. NbRdR6 and NbSGS3 or SGS3 domains were cloned into pGADT7 vector using Gibson assembly or with Gateway cloning strategies. For yeast three-hybrid experiments, Coremin and Nimeroc sequences were expressed through chimera RNAs by pIIIMS2.1 plasmid [22] and proteins fused to myc tag in pGAD-T7 plasmids using *Eco*RI and *Xma*I restriction sites. Selective markers for pGBK, pGAD and pIIIMS2 are tryptophan, leucine and uracil, respectively. For pull-down assays, NbDRB4 and EGFP were N-terminally fused to His-tag, while p14 (and hypomorphic mutant *p14BA2*) were N-terminally fused to MBP-tag in pETc backbone vector by Golden Gate assembly. Primers used in the study are presented in Supplementary Table S1.

### 2.4. Agroinfiltration

Cultures of *Agrobacterium tumefaciens* (strain GV3101) were prepared in LB media supplemented with kanamycin (100 µg/mL), rifampicin (100 µg/mL) and gentamicin (30 µg/mL) and incubated at 28 °C for 18 h. Cells were centrifuged, resuspended and incubated for at least two hours in acetosyringone (200 µM) and MgCl_2_ (10 mM). Leaves of 5-week-old WT or RdR6i *N. benthamiana* were (co-)infiltrated with bacteria suspension using individual optical density 600 nm set to 0.5 or to 1 for TurboID.

### 2.5. Generation of Stable N. benthamiana Transgenic Lines

Stable transgenic *Nicotiana benthamiana* plants expressing NbDRB4-tRFP, AtDRB4-tRFP, or tRFP alone were generated by *Agrobacterium tumefaciens*-mediated transformation. Leaves were agroinfiltrated with the corresponding pEAQ constructs and harvested 3 days post-infiltration. Leaf tissues were surface-sterilized in 4% sodium hypochlorite for 20 min and rinsed three times with sterile water for 10 min. Leaf discs were excised and cultured on Murashige and Skoog (MS) medium (Duchefa^®^, Haarlem, The Netherlands) supplemented with 3% (*w*/*v*) sucrose, 0.8% (*w*/*v*) agar, 2 mg L^−1^ 6-benzylaminopurine (BAP), 0.05 mg L^−1^ α-naphthaleneacetic acid (NAA), 150 mg L^−1^ kanamycin, and 500 mg L^−1^ carbenicillin (pH 5.8). Cultures were maintained at 25 °C under a 16 h light/8 h dark photoperiod. Calli were transferred onto fresh selection medium every 15 days until shoot regeneration. Regenerated shoots were transferred to rooting medium containing half-strength MS salts, 1.5% (*w*/*v*) sucrose, and 0.8% (*w*/*v*) agar (pH 5.8). After root development, plantlets were acclimatized and transferred to soil.

### 2.6. Silencing Suppression Assay

Silencing suppression activity using GFP and GF-FG trigger assay was performed according to [23].

### 2.7. Immunoprecipitation

Agroinfiltrated *N. benthamiana* leaves expressing p14 or Flag-p14 were harvested at 3 days post-infiltration. An amount of 100 mg of leaf sample was ground in liquid nitrogen and homogenized for 10 min in 1 mL of lysis buffer (50 mM Tris-HCl (pH 7), 50 mM NaCl, 0.1% Triton, 1 mM DTT, supplemented with one tablet of cOmplete™ protease inhibitor cocktail for 50 mL of buffer) at 12 rpm at 4 °C. Cellular debris was removed by two successive centrifugations at 16,000× *g* for 10 min at 4 °C. Supernatants were incubated at 4 °C with magnetic beads conjugated to an anti-Flag monoclonal antibody (µMACS system, Miltenyi Biotech, Bergisch Gladbach, Germany) for 30 min at 6 rpm. Samples were dropped on µColumn, then washed with 1 mL of lysis buffer and with 100 µL of washing buffer (20 mM Tris-HCl (pH 7.5)). Elution was performed with 90 °C pre-warmed elution buffer (50 mM Tris-HCl (pH 6.8), 50 mM DTT, 1% SDS, 1 mM EDTA, 0.005% bromophenol blue, 10% glycerol), and samples were analysed by liquid chromatography coupled to tandem mass spectrometry (LC-MS/MS).

### 2.8. TurboID Proximity-Labelling

Agroinfiltrated *N. benthamiana* leaves expressing TurboID-EYFP or TurboID-EYFP-p14 were infiltrated 40 h later with a solution of 200 µM biotin (in 10 mM MgCl_2_). Four infiltrated leaves from each plant were then harvested after 6 h incubation and flash-frozen in liquid nitrogen. For each TurboID fused construct, three biological replicates were performed. Specifically, 1 g of leaf samples were ground to a fine powder in a mortar and homogenized for 5 min with 2 mL of RIPA lysis buffer (50 mM Tris-HCl (pH 7.5), 150 mM NaCl, 0.5% sodium deoxycholate, 1% Triton X-100, 0.1% SDS, supplemented with one tablet of cOmplete™ protease inhibitor cocktail (Roche, Basel, Switzerland) for 50 mL of buffer) by vortexing. Lysates were sonicated three times for 15 s using a Bioruptor^®^ (Diagenode, Seraing, Belgium) with 30 s breaks, and cellular debris was removed by centrifugation at 4000 rpm for 10 min at 4 °C. Moreover, cellular debris was once again removed by two successive centrifugations at 20,000× *g* for 10 min at 4 °C. The clear supernatant was applied to Zeba™ Spin Desalting (Thermo Scientific™, Illkirch Graffenstaden, France) column to remove excess free biotin using the gravity protocol according to the manufacturer’s instructions. Briefly, the storage solution was eliminated from the column by 2 min centrifugation at 1000× *g* and then equilibrated with 5 mL of RIPA buffer. Samples were loaded onto the column and centrifugated for 2 min at 1000× *g*. The protein concentration of the extract was then measured by Qubit Protein Assay (Thermo Scientific). A quantity of 10 mg of protein lysates was incubated at 4 °C with 100 µL of pre-washed DynaBeads™ MyOne™ Streptavidin-C1 magnetic beads (Invitrogen™, Illkirch Graffenstaden, France) overnight on a rotor wheel. The next day, the beads were separated from the protein extract on a magnetic rack and washed with 1 mL of each of the following solutions by incubating on a rotor wheel for 5 min and removing the wash solutions: onetime with cold RIPA buffer, one time with cold 1 M KCl, one time with cold 100 mM Na_2_CO_3_, one time with 2 M Urea (in 10 mM Tris-HCl pH 8) at room temperature, two times with cold RIPA buffer (without protease inhibitor) and finally five times with 50 mM NH_4_HCO_3_, before being resuspended in 100 µL of NH_4_HCO_3_ and analysed by liquid chromatography coupled to tandem mass spectrometry (LC-MS/MS) analysis.

### 2.9. Mass Spectrometry Analysis and Data Processing

Biotinylated proteins enriched with streptavidin beads were digested into peptides via on-bead digestion by sequencing-grade porcine trypsin (Promega) and analyzed on a TIMS TOF pro2 mass spectrometer coupled to a nanoElute 2 (Bruker) using a data-dependent acquisition strategy by the Strasbourg-Esplanade Proteomics Facility at the Institut de biologie moléculaire et cellulaire (IBMC). The raw data were searched against the *N. benthamiana* sequences (SOL Genomics website, v.1.0.1, https://solgenomics.net/ftp/genomes/Nicotiana_benthamianaV261/, accessed 13 July 2025) and a database containing the BNYVV p14, streptavidin and TurboID sequences. Peptides were identified with Mascot algorithm (version 2.8, Matrix Science, London, United Kingdom), imported into Proline v2.1.2 software [24] and validated with a false discovery rate of <1% at the peptide spectrum matches and protein levels. Statistical analysis of the protein abundances was carried out with Prostar software (version 1.36.1 [25]) to normalize, impute missing values (Det quantile 1%) and calculate p-values adjusted by the Benjamini–Hochberg method, as well as a protein fold change. The results are presented in a Volcano plot generated in R (v4.4.2) using protein log2 fold changes and their corresponding adjusted p-value (-log10adjp) to highlight enriched and depleted proteins. Data are available via ProteomeXchange with identifier PXD068024.

### 2.10. Plant Infection

BNYVV infections were triggered by mechanical inoculation of RNA1, RNA2 (WT, 2BA2 or 2BA3) and RNA3 or RNA3E in vitro transcripts. A quantity of 10 µg of RNA1 and 5 µg of RNA2 and RNA3 (or RNA3E) were necessary to infect one 4-week-old *N. benthamiana*. Nuclease free inoculum was prepared in 100 µL of 50 mM KH_2_PO_4_ (pH 7.5) containing 0.05% macaloïd.

### 2.11. Yeast Two- and Triple-Hybrid Experiments

All yeast procedures were performed on *Saccharomyces cerevisiae* as described previously for yeast two-hybrid [26] and yeast three-hybrid [27].

### 2.12. Pull-Down Assay

The constructs His-EGFP, His-DRB4, MBP, MBP-p14 and MBP-*p14BA2* were individually transformed into *E. coli* BL21 (DE3) competent cells. The transformed cells were grown at 37 °C to OD_600_ of 0.6–0.8. Then each protein was induced with 0.5 mM isopropyl-β-D-thiogalactopyranoside (IPTG) for 5 h at 25 °C. The resulting cultures were centrifuged and resuspended in ice-cold lysis buffer specific to each fusion tag: for MBP-fused proteins (20 mM HEPES [pH 7.9], 200 mM KCl, 1 mM DTT, 1X cOmplete Protease Inhibitor Cocktail) and for His-fused proteins (50 mM Tris-HCl [pH 8], 300 mM NaCl, 30 mM imidazole, 5% glycerol, 1 mM DTT, 1X COmplete Protease Inhibitor Cocktail). His-tagged recombinant proteins were purified by using a high-affinity Ni^2+^-NTA resin (Macherey-Nagel, Düren, Germany) and eluted with 300 mM imidazole. The induction and elution of proteins were verified by SDS-PAGE electrophoresis. MBP-tagged recombinant proteins were immobilized on amylose resin (New England Biolabs, Ipswich, MA, USA) and incubated with purified His-EGFP or His-DRB4 for 2 h at room temperature. Following three washes with the corresponding lysis buffer, proteins were eluted by directly adding 2X Laemmli to the resin. Interaction was analysed by SDS-PAGE followed by immunoblotting using anti-His antibodies.

### 2.13. Protein and RNA Extraction and Analyses

Leaves were ground in liquid nitrogen to obtain a fine powder, and except for subcellular fractionation and small RNA analysis, 100 mg and 200 mg were harvested for protein and RNA extraction, respectively. Samples were mixed vigorously with glass beads in Precellys^®^ in a “polysome” buffer (200 mM Tris-HCl (pH 9), 400 mM KCl, 200 mM saccharose, 35 mM MgCl_2_, 25 mM EGTA) to extract RNAs using a phenol/chloroform purification. Nucleic acids were precipitated with ethanol. A quantity of 200 µL of 3 M NaAc were added on pellet to solubilize small RNAs and DNA. Remaining RNA pellet was washed with 70% ethanol and then dissolved in 50 to 100 µL of nuclease free water, depending on the pellet size. RNA quantity was estimated using spectrophotometry and quality estimated on agarose gel. High-molecular-weight Northern blot was performed using 5 µg of RNA, mixed with 4 volumes of HFF buffer (20 mM HEPES-KOH (pH 7.8), 1 mM EDTA, 50% formamide, 6% formaldehyde, 0.005% bromophenol blue). Sample denaturation was performed for 5 min at 65 °C before loading on denaturing agarose gel (1% agarose, 20 mM HEPES-KOH (pH 7.8), 1 mM EDTA, 6% formaldehyde). After migration in HEPES-EDTA buffer, RNAs were transferred on Hybond™ membrane in 20X SSC for 12 h. RNA were UV crosslinked and membrane stained with methylene blue. Pre-hybridization was performed in 50% formamide, 5X SSC, 8X Denhardt, 50 mM NaH_2_PO_4_/Na_2_HPO_4_ (pH 6.5), 0.1% SDS solution for radiolabelled RNA probes or in PerfectHyb™ Plus Hybridization buffer for radiolabelled DNA probes. A 5′RACE experiment was performed as described previously [15]. Briefly, 1 µg of total RNAs treated with DNase I were ligated, if they possessed a 5′-monophosphorylated extremity, with an oligoribonucleotide rRACE-5 (5′-GAACACUGCGUUUGCUGGCUUUGAUGAAA-3′) using T4 RNA ligase (New England Biolabs) before a reverse-transcription step using T12R3 primer (5′-AAGCTTTTTTTTTTTTGTCAATATACTG-3′) and SuperScript IV (Invitrogen™) reverse transcriptase. Detection of ncRNA3 was performed by PCR amplification using RACE-5 primer (5′-GAACACTGCGTTTGCTGG-3′) and a specific anti-sense primer (5′-GGACTGGTACATTTCACACCC-3′). Amplicons were cloned into pUC19 and sequenced to characterize their 5′ extremity. Nucleic acids were phenol/chloroform purified and precipitated after each treatment.

For protein analyses, samples were directly ground in 2X Laemmli buffer and boiled for 2 min at 90 °C. Following 1 min at 12,000× *g* centrifugation, supernatant was loaded on a polyacrylamide gel in Tris-glycine buffer. Proteins were transferred on Immobilon-P PVDF membrane in Tris-ethanol buffer and incubated with antisera directed against BNYVV or GFP proteins or with monoclonal antibody Streptavidin fused to HRP. Following washes in 1X PBS, 0.5% SDS, HRP conjugated goat anti-rabbit secondary antibody was used to detect protein using “Lumi-light Western blotting substrate” (Roche) and visualized thanks to Fusion FX (Vilber). Membranes were further stained with Coomassie blue to check for equal loading.

### 2.14. Microscopy

#### 2.14.1. Localization of Fluorescent Labelled Proteins Using Confocal Microscopy

Agroinfiltrated leaf disks were analysed for 3 days or 40 h (for TurboID) post-agroinfiltration. Confocal laser scanning microscopy (CLSM) was performed with a Zeiss LSM700 (ZEISS, Oberkochen, Germany) or Leica SP8 microscope (Leica, Wetzlar, Germany). EGFP, EYFP and tRFP were excited at 488 nm, 514 nm and 555 nm, respectively. Samples were observed using a 40x magnification under oil immersion. Image acquisitions were performed with ZEN v3.11 (ZEISS, Oberkochen, Germany) or LasX software v3.11.0 (Leica, Wetzlar, Germany) and further edited with ImageJ v1.54m.

#### 2.14.2. FRET-FLIM

Lifetime of eGFP fusion proteins was measured in the presence of tRFP alone as a control and of NbDRB4-tRFP. Acquisitions were performed on Nikon TE2000 microscope (Nikon, Amstelveen, The Netherlands) on leaf disks three days post-agroinfiltration.

### 2.15. Plant Subcellular Fractionation

A total of 0.5 g of *N. benthamiana* infected leaves were ground in liquid nitrogen and 10 mL of resuspension buffer n°1 (0.4 M sucrose, 10 mM Tris-HCl (pH 7.5), 10 mM MgCl_2_, 2.5 mM DTT) added before two filtrations on Miracloth. Following centrifugation for 20 min at 3200× *g*, supernatants were harvested for cytosolic protein and RNA analyses. Pellets were resuspended in 1 mL of resuspension buffer n°2 (0.25 M sucrose, 10 mM Tris-HCl (pH 7.5), 10 mM MgCl_2_, 1% Triton, 2.5 mM DTT) and centrifuged for 10 min at 16,000× *g*. Supernatants were gently discarded and pellets resuspended in 300 µL of resuspension buffer n°2 and carefully settled on 300 µL on sucrose cushion (1.75 M sucrose, 10 mM Tris-HCl (pH 7.5), 2.5 mM MgCl_2_, 0.15% Triton, 2.5 mM DTT). Samples were centrifuged for 1 h at 16,000× *g*. Nuclei present in the pellets were resuspended in 200 µL of lysis buffer (50 mM Tris-HCl (pH 7.5), 10 mM EDTA, 1% SDS). For analyses, 5 mL and 100 µL of cytosolic and nuclear fractions were used respectively to perform TRIzol™ extraction following manufacturer protocol. A DNase treatment was performed on extracted RNAs from nuclei.

## 3. Results

### 3.1. Identification of p14 Potential Partners

**Figure 1:**
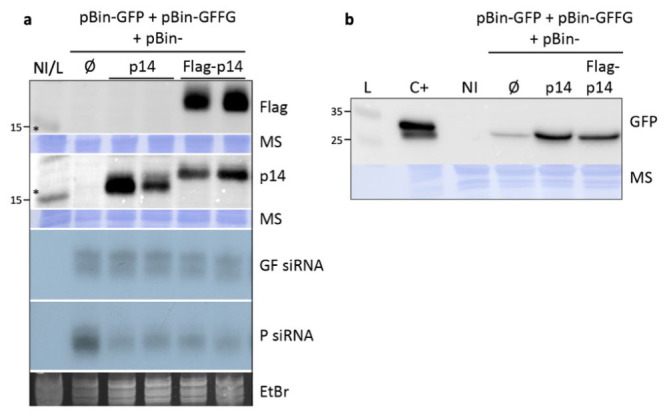
Flag-p14 is a functional version of p14 in a GFP reporter assay. *N. benthamiana* leaves were co-infiltrated with agrobacterium carrying pBin-GFP, the silencing trigger pBin-GFFG and control vector, pBin-Ø or VSR expressing vector pBin-Flag-p14. Small RNAs and proteins were extracted with TRIzol™ from agroinfiltrated leaves. Small RNAs were subjected to low-molecular-weight Northern blot analysis. GF and P siRNAs were detected using probes specific to the 5′ and 3′ parts of the GFP mRNA, respectively. Western blot analysis of (Flag)p14 (**a**) and GFP (**b**) were performed using specific antibodies. NI, non-infiltrated; L, ladder; MS, membrane staining; EtBr, ethidium bromide; C+, GFP positive control. In (**a**), a non-infiltrated leaf extract was mixed with the ladder (NI/L), and asterisks (*) represent the 15 kDa protein of the ladder.

To further investigate the p14 VSR function and identify potential interacting partners, we first performed immunoprecipitation (IP) assays and later a proximity labelling experiment using TurboID, both coupled to mass spectrometry. For this purpose, we used protein extracts from *N. benthamiana* leaves overexpressing a tagged version Flag-p14. Functionality of the Flag-p14 was verified by comparing its silencing suppression activity to the p14 WT protein using the GFP reporter gene and the GF-FG trigger in an assay previously described [23]. Both p14 and Flag-p14 proteins were similarly expressed and induced a comparable reduction in secondary siRNAs (Figure 1a, panel p14 and panel P siRNA) restoring GFP protein accumulation (Figure 1b, right panel). Thus, Flag-p14 is a functional version of the p14 protein and can be used for immunoprecipitation experiments. Immunoprecipitations were performed using anti-Flag monoclonal antibodies, and the resulting samples were analysed by mass spectrometry. A majority of ribosomal proteins were retrieved in IP experiments. The relevance of those translational-associated proteins has already been described during viral infection (for review, see [28]). However, ribosomal proteins remain difficult to study, particularly in *N. benthamiana* where mutant lines and genetics tools are not easily available, nor engineered. We therefore focused our analyses on immunoprecipitated candidates related to the RNA silencing pathway. Among the candidates, DRB4 was identified in one experiment (Supplementary Figure S1), without retrieval in the other experiments. Although its homolog in *A. thaliana* (AtDRB4) has already been described in silencing pathways [11], we decided to study this candidate (NbDRB4) to unravel its function in *N. benthamiana*.

### 3.2. A New NbDRB4 cDNA Was Obtained and Its Product Localizes in the Nucleolus

Four distinct DRB4 sequences are annotated in the *N. benthamiana* genome (Supplementary Figure S2). Although DRB4 from *N. benthamiana* has been previously studied by Barton et al. and Fátyol et al. [29,30], sequence alignment analysis revealed that their described DRB4 sequences do not correspond to either Niben101Scf06376g01010 or Niben101Scf06376g01014 encoded NbDRB4 (Supplementary Figure S2). Actually *N. benthamiana* possesses a tetraploid genome resulting from an interspecific hybridization event between *Nicotiana sylvestris* and *Nicotiana noctiflora* [31]. Despite multiple genome rearrangements, it seems that *N. benthamiana* has two putative homologs of DRB4: the previously annotated Niben101Scf05841g01021 as reported in earlier studies, and Niben101Scf06376g01010 newly identified in this study. Following mass spectrometry results, we identified Niben101Scf06376g01010 and Niben101Scf06376g01014 peptides spectra (Supplementary Figure S1). Total RNAs have been extracted from mock and infected leaf samples and subjected to RT-PCR but we only succeeded in Niben101Scf06376g01010 cDNA obtention.

**Figure 2:**
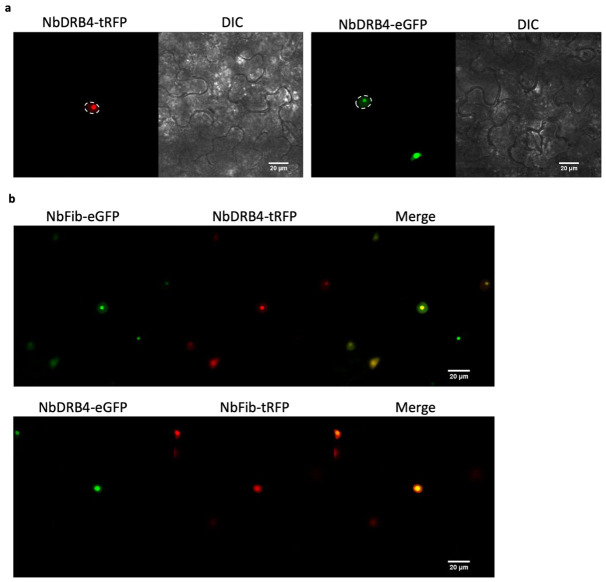
NbDRB4 localizes in the nucleolus in *N. benthamiana*. Transiently expressed NbDRB4 fused to tRFP (**left panel**) or eGFP (**right panel**) (**a**) colocalized with fibrillarin fused to eGFP (**upper panel**) or tRFP (**lower panel**) (**b**). Nuclei are surrounded by dashed lines and visualized in differential interference contrast (DIC) in (**a**). Observations were performed three days post-infiltration on a confocal microscope. Scale bars are indicated.

NbDRB4 sequence was cloned in fusion with eGFP or tRFP within an overexpression vector and the following NbDRB4-eGFP and NbDRB4-tRFP both localized in nuclei and likely in nucleolus (Figure 2a). To confirm such nucleolar localization, we cloned the *N. benthamiana* fibrillarin fused to eGFP or tRFP and coexpressed these proteins with the other tagged version of NbDRB4. Whatever the combination tested, both NbDRB4 and fibrillarin proteins clearly colocalized, confirming that NbDRB4 is mostly present in the nucleolus and partially diffuse in the nucleoplasm (Figure 2b).

### 3.3. Overexpression of NbDRB4 Relocalizes p14 to the Nucleolus Without Detectable Direct Interaction and Independently of RDR6 Activity

**Figure 3:**
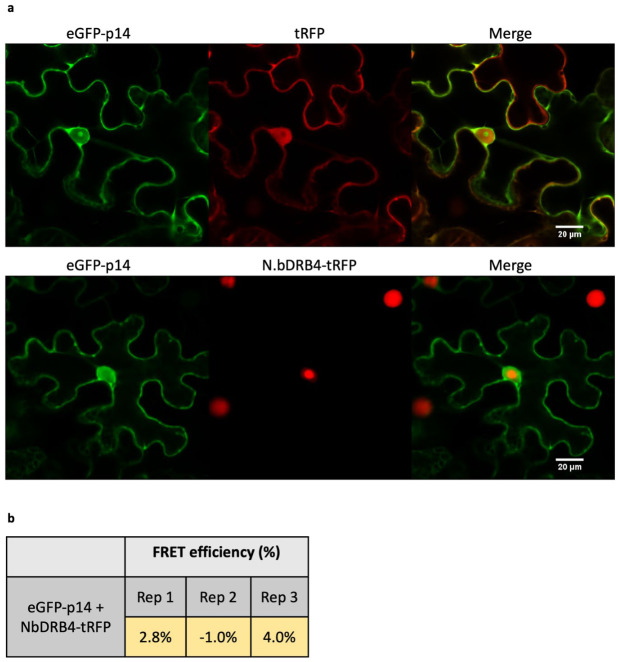
Colocalization and interaction assay of eGFP-p14 and NbDRB4-tRFP. (**a**) Transiently expressed eGFP-p14 and tRFP (**upper panel**) or NbDRB4-tRFP (**lower panel**). Observations were performed three days post-infiltration on a confocal microscope. Scale bars are indicated. (**b**) FRET efficiency (%) was determined for three independent experiments (Replicate, Rep). The absence of interaction is highlighted in yellow background.

Based on the mass spectrometry results, it was necessary to validate the interaction between p14 and NbDRB4. Taking advantage of fluorescent proteins, a FRET-FLIM approach was chosen using tRFP as a control. The eGFP lifetime measurements were performed following confocal laser microscopy observations to verify co-expression of both fusion proteins within the same cells. Protein accumulation levels analysed by Western blot confirm that eGFP-p14, NbDRB4-tRFP and tRFP were correctly expressed (Supplementary Figure S3a, left panel). Expressed alone, the eGFP-p14 localized to the nucleolus and diffused in both the nucleoplasm and the cytoplasm ([13]; Figure 3a) and was not altered in the presence of tRFP. Interestingly, when co-expressed with NbDRB4-tRFP, eGFP-p14 was excluded from the nucleolus. The interaction between p14 and NbDRB4 seems therefore unlikely but has been nevertheless investigated in whole nuclei. Over three experiments, the interaction between p14 and NbDRB4 has been tested in 75 nuclei and FRET efficiency was always below the conventional 7% threshold fixed to consider an effective interaction (Figure 3b). In parallel, localization and interaction between eGFP-p14 and AtDRB4-tRFP were studied. AtDRB4-tRFP was mainly nuclear but excluded from nucleoli and did not colocalize nor interact with eGFP-p14 (Supplementary Figure S4).

**Figure 4:**
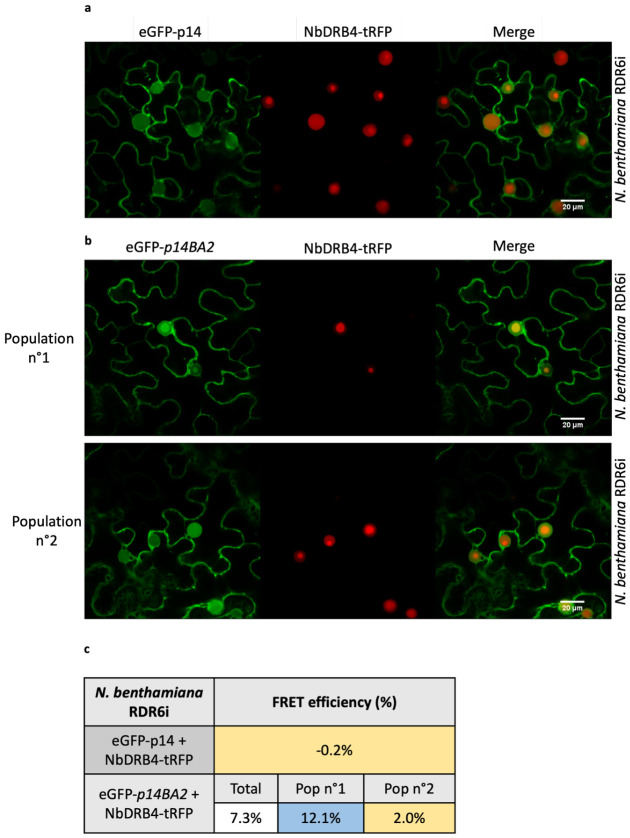
Effect of RDR6 knockdown on eGFP-p14 and eGFP-*p14BA2* localizations and their interaction with NbDRB4-tRFP in *N. benthamiana RdR6i*. (**a**) Transiently expressed eGFP-p14 and NbDRB4-tRFP (**upper panel**) or (**b**) eGFP-*p14BA2* and NbDRB4-tRFP (**lower panels**). Observations are performed three days post-infiltration. Scale bars are indicated. (**c**) FRET efficiencies (%) are depicted with colour codes. The yellow and blue backgrounds highlight the absence and the presence of interaction, respectively. For eGFP-*p14BA2*, mean eGFP lifetime is indicated in the “Total” column and split values in columns Pop n°1 (*n* = 9) and n°2 (*n* = 10).

BNYVV VSR affects the RNA silencing transitivity pathway, reducing secondary siRNAs. The mutation of the p14BA2 protein impairs its silencing suppression activity but maintains the protein subcellular localization in the nucleolus and in the cytoplasm [13]. Within an RDR6 knockdown context (RdR6i *N. benthamiana*), the mutant expressing the hypomorph p14BA2 protein behaves as the wild-type virus and moves systemically [16]. However, nothing was known about the subcellular localization of p14 and p14BA2 proteins in the RdR6i context. Expressed alone or in combination with tRFP in RdR6i *N. benthamiana*, eGFP-p14 localized similarly as in wild-type (WT) plants and in the presence of NbDRB4-tRFP, p14 was excluded from the nucleolus (Figure 4a). When FRET efficiency was analysed in RdR6i *N. benthamiana*, no interaction was observed. We then tested the behaviour of *p14BA2* mutant in the presence of NbDRB4-tRFP. The eGFP-*p14BA2* mutant was localized as eGFP-p14 in WT plants ([13] and this study). However, two localization profiles were observed in RdR6i plants when co-expressed with NbDRB4-tRFP. One remained nucleolar and colocalized with NbDRB4-tRFP (population n°1, Figure 4b), and the other was similar to p14 with a reduced nucleolar localisation (population n°2, Figure 4b). While fusion proteins were homogeneously expressed in RdR6i *N. benthamiana* plants (Supplementary Figure S5), two distinct FRET efficiencies pools were determined. The first one was higher than the 7% threshold revealing a direct interaction between *p14BA2* and NbDRB4 (Figure 4c), and the second was lower, indicating an absence of interaction. Following these results, we decided to perform the same analysis in WT *N. benthamiana* plants.

### 3.4. p14BA2 Hypomorphic Mutant Colocalizes and Interacts with NbDRB4

**Figure 5:**
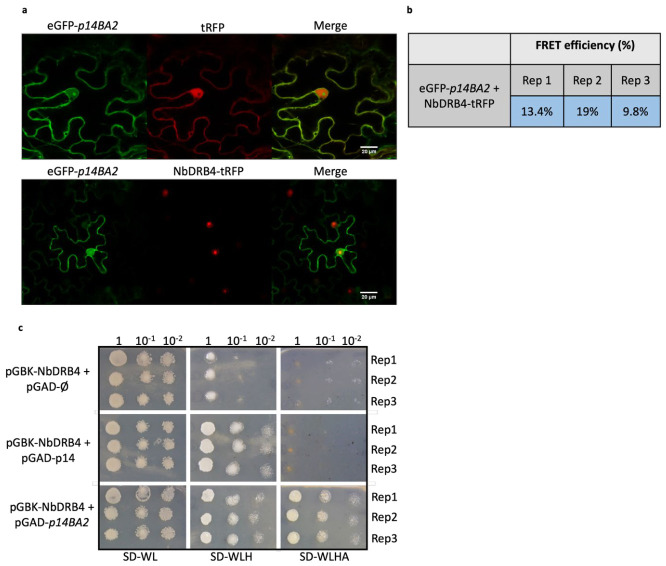
Colocalization and interaction assay of eGFP-p14BA2 and NbDRB4-tRFP by Y2H. (**a**) Transiently expressed eGFP-*p14BA2* and tRFP (**upper panel**) or NbDRB4-tRFP (**lower panel**) were observed three days post-infiltration in *N. benthamiana* on a confocal microscope. Scale bars are indicated. (**b**) FRET efficiencies (%) were determined for three independent experiments. Interaction is highlighted by a blue background. (**c**) Interaction between p14 and *p14BA2* and NbDRB4 is studied by yeast two-hybrid on selective SD-WLH and selective and stringent SD-WLHA media. Rep, replicate; SD, synthetic defined; W, tryptophan; L, leucine; H, histidine; A, adenine.

The *p14BA2* protein was colocalized in the nucleolus when expressed with NbDRB4 (Figure 5a, lower panel). Proteins were similarly expressed in *N. benthamiana* (Supplementary Figure S3a, right panel). EGFP lifetime measurements performed on 68 nuclei across three independent experiments revealed a detectable interaction between *p14BA2* and NbDRB4 (Figure 5b) with FRET efficiency ranging from 9.8 to 19%. This interaction was further confirmed using yeast two-hybrid (Y2H) experiments (Figure 5c). Yeast growth was observed for p14 WT on SD-WLH but abolished on stringent SD-WLHA media, suggesting a weak interaction between p14 and NbDRB4. Given that fibrillarin was described as a key player in many viral long-distance movements [32], we investigated whether this protein was able to interact either with p14 proteins or NbDRB4. However, no direct interaction was observed by FRET between fibrillarin and NbDRB4 or any of the p14 proteins (Supplementary Figure S6).

**Figure 6:**
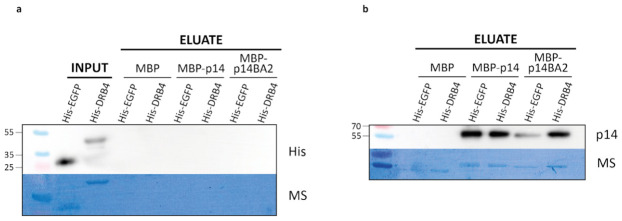
Analysis of the interaction between p14(BA2) and NbDRB4 in vitro by MBP-pull down assay. The purified His-tagged NbDRB4 or EGFP were incubated with MBP-tagged p14 or mutant *p14BA2*. After being pulled-down with amylose resin, the proteins were detected by Western blot with anti-His (**a**) or anti-p14 (**b**) antibodies. MS, membrane staining.

To further assess the potential interaction between p14 and NbDRB4, we performed in vitro pull-down assays using recombinant maltose binding protein MBP, MBP-p14, and MBP-*p14BA2* proteins expressed in *E. coli*, along with His-NbDRB4 as putative binding partner and His-EGFP as a negative control. Although both MBP-p14 and MBP-*p14BA2* were efficiently pulled down (Figure 6b), in contrast to the interaction observed in vivo, no binding to His-DRB4 was detected under the conditions tested (Figure 6a).

### 3.5. p14BA2 and NbDRB4 Interaction Is Specifically Abolished by ncRNA3 Accumulation

**Figure 7:**
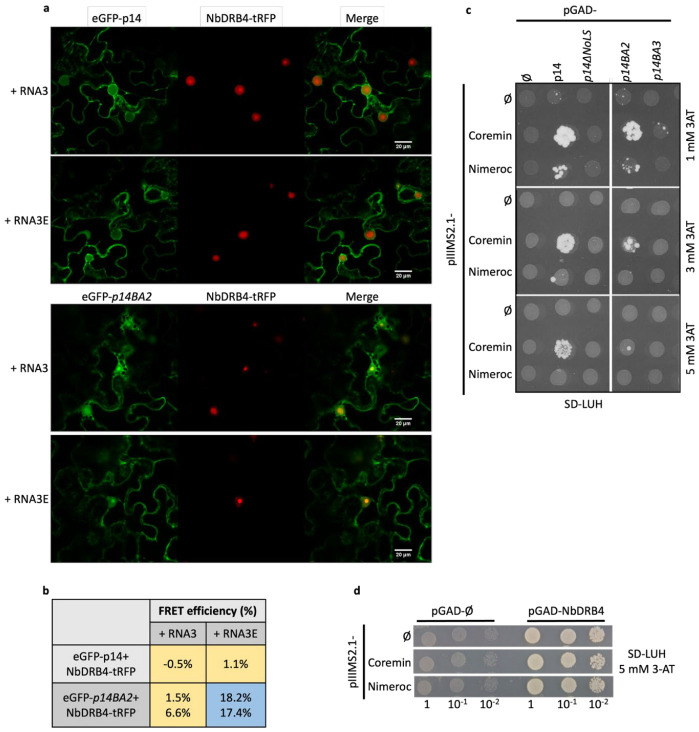
Effect of RNA3 and RNA3E on p14 and *p14BA2* subcellular localization and their interaction with NbDRB4. (**a**) Transiently expressed eGFP-p14 (**upper panel**) or eGFP-*p14BA2* (**lower panel**) and NbDRB4-tRFP in the presence of RNA3 or RNA3E. Observations were performed three days post-infiltration on a confocal microscope. Scale bars are indicated. (**b**) FRET efficiencies (%) are depicted with colour code; the yellow and blue backgrounds depict the absence and the presence of interaction, respectively. (**c**,**d**) Yeast three-hybrid interaction analyses between Coremin or Nimeroc sequences with p14, *p14∆NoLS, p14BA2* and *p14BA3* (**c**) or NbDRB4 (**d**). Interaction was tested on selective SD-LUH media, supplemented with 5 mM of 3-AT. SD, synthetic defined; L, leucine; U, uracil; H, histidine; 3-AT, 3-Amino-1,2,4-triazole.

The virus carrying the *p14BA2* mutation is unable to establish a systemic infection on its own while its systemic spread is restored in the presence of an efficient ncRNA3 accumulation [16]. We studied the effect of ncRNA3 accumulation on the subcellular localization of *p14BA2* and its interaction with NbDRB4. WT RNA3 and therefore ncRNA3 accumulation and the negative control RNA3E unable to accumulate ncRNA3 were expressed from a 35S-driven cDNA clone. The production of RNA3 or RNA3E did not alter the localization of eGFP-p14, which remained excluded from the nucleolus in the presence of NbDRB4 (Figure 7a). Conversely, the eGFP-*p14BA2* fusion protein was localized in the nucleolus regardless of the RNA3 species present (Figure 7a). FRET-FLIM analyses revealed that eGFP-*p14BA2* was not able to interact with NbDRB4-tRFP in the presence of ncRNA3 (FRET efficiencies of 1.5% and 6.6%; Figure 7b), behaving as wild-type p14. On the contrary, in the absence of ncRNA3, the interaction remained (FRET efficiencies of 18.2% and 17.4%; Figure 7b). Proteins and RNA accumulation levels were verified by Western blot (Supplementary Figure S3a) and Northern blot (Supplementary Figure S3b), respectively. ncRNA3 accumulated in the presence of tRFP and either eGFP-p14 or eGFP-*p14BA2*. Interestingly, a reduced accumulation of ncRNA3 was observed when NbDRB4-tRFP was co-expressed, which correlated with an increased level of its precursor (RNA3) (Supplementary Figure S3b, see arrows). RNA3 and RNA3E differ by a 20 nts Coremin sequence that is revert-complemented (in RNA3E namely nimeroc). According to unpublished data by K. Hleibieh [33] (Figure 7c), a specific interaction occurs between the Coremin sequence and p14, p14BA2 but not with p14BA3 or the ∆NoLS mutant described by [13]. This prompted us to investigate whether NbDRB4 could contribute to this specificity. Yeast three-hybrid experiments revealed that the NbDRB4 fusion protein exhibits non-specific RNA binding activity (Figure 7d), suggesting that ncRNA3 affects the VSR per se, rather than through a specific interaction with NbDRB4.

### 3.6. NbDRB4-tRFP Overexpression Induces Developmental Issues Due to Silencing Suppression Activity

Transgenic *N. benthamiana* plants were created to express NbDRB4- and AtDRB4-tRFP. Plants expressing AtDRB4-tRFP were successfully obtained, while those producing NbDRB4-tRFP displayed severe dwarfism (Supplementary Figure S7a) and were sterile despite ovules and pollen having an identical phenotype to the tRFP control plant (Supplementary Figure S7b). This observation is particularly striking because a virus-induced gene silencing (VIGS) approach targeting NbDRB4 did not lead to any phenotype on silenced plants. These transgenic plants would have been of particular interest to perform confocal observations and FRET-FLIM experiments, particularly using the BNYVV replicon strategies [34] to produce fluorescent proteins in the viral context. Transient expression of the constructs in a reporter system revealed a potential silencing suppressor activity when NtDRB4-tRFP (and AtDRB4-tRFP) was used. Similar accumulation levels of GFP and siRNA were observed for AtDRB4 (Supplementary Figure S8), whereas this protein is not described as an endogenous silencing suppressor but is rather involved in antiviral defence mechanisms.

### 3.7. RNA3 and ncRNA3 Are Differentially Localized in Infected Tissues

**Figure 8:**
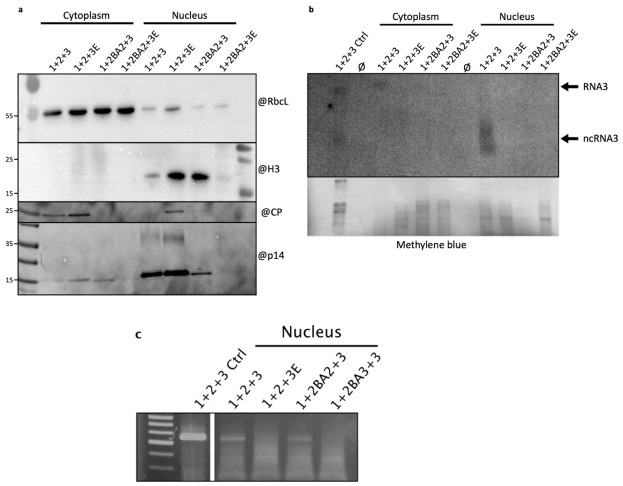
Genomic and non-coding RNA3 display different cellular localization. *N. benthamiana* were infected with RNA1, RNA2 or RNA2 expressing *p14BA2* (2BA2) or *p14BA3* (2BA3) and RNA3 or RNA3E. Apical leaves were subjected to cellular fractionation. (**a**) Cytosolic and nuclear fractions qualities were controlled through anti-RbcL and anti-H3 detection by Western blots. Viral infections and p14 accumulation levels were analysed with @CP and @p14 antibodies. Molecular weights (kDa) are indicated on the left. (**b**) RNA3 and ncRNA3 were detected by Northern blot using riboprobe targeting the 3′ extremity of ncRNA3. (**c**) ncRNA3 accumulation was detected by 5′RACE experiment. Nuclear and total RNA were loaded on two separate agarose gels. Non-fractionated plant sample infected with RNA1, RNA2 and RNA3 (1+2+3 Ctrl) was used as a control.

BNYVV replicates in the cytoplasm where its messenger RNAs accumulate for their expression, replication and encapsidation. If some BNYVV proteins such as p25 and p14 have been detected in the nuclear compartment, viral RNAs are expected to be excluded from the nucleus. The NbDRB4•*p14BA2* nucleolar interaction was disrupted in the presence of Coremin containing RNAs. Therefore, we analysed the presence of RNA3 and ncRNA3 in cellular fractions collected from *N. benthamiana* plants 20 days post-infection. Fractionation quality was evaluated by immunodetection of the cytoplasmic large rubisco subunit (RbcL) and the nuclear Histone H3 (Figure 8a). Immunodetection of the coat protein (CP) confirmed successful plant infection and revealed CP protein accumulation in the cytoplasm (Figure 8a). However, the CP signal observed in the nuclear 1+2+3E sample indicated cytoplasmic contamination which was corroborated by the @RbcL Western blot (Figure 8a). Detection of p14 highlighted that plant systemic infection occurred with the virus expressing *p14BA2* supplemented with RNA3 [16]. Both p14 and p14BA2 were detected in the cytoplasm and nucleus (Figure 8a). Viral RNA levels from plant infected with the BA2 mutant were below the detection limit (Figure 8b), whereas systemic movement was observed in the presence of RNA3, as indicated by the detection of p14 (Figure 8a). RNA3 and ncRNA3 were detected in the plant infected with wt RNAs (Figure 8a, 1+2+3 Ctrl). While genomic RNA3 was slightly observed in the cytoplasm fraction, ncRNA3 did highly accumulate in the nucleus (Figure 8b, 1+2+3 Nucleus), with no significant presence in the cytoplasm. As previously described [17], RNA3E is unable to produce ncRNA3 (Figure 8b, 1+2+3E Nucleus), but surprisingly, RNA3E was not detected in the cytosolic fraction (Figure 8b, 1+2+3E Cytoplasm). Perhaps this was due to experimental limitations, as only 1 µg of RNA was loaded for Northern blot analyses. This limitation may certainly explain the difficulty of obtaining evidence of RNA3 accumulation. To bypass problems linked to p14BA4 and RNA3E accumulation levels and explore the role of p14 in the subcellular localization of ncRNA3, an additional subcellular fractionation was performed, including the nucleo-cytoplasmic p14-BA3 mutant (K^82^A-K^86^A) which is unable to bind Coremin (Figure 7c), but is competent for systemic spread [13,33]. After confirming infection via detection of the CP and p14 (Supplementary Figure S9a), we performed a 5′ rapid amplification of cDNA ends (5′RACE) targeting ncRNA3 on nuclear samples [15]. We observed a specific signal for ncRNA3 in the nuclear fraction of plants infected with RNA1+2+3 and RNA1+2BA2+3 (Figure 8c). In contrast, no specific signal was detected in plants infected with RNA1+2BA3+3 expressing the *p14BA3* mutant unable to bind Coremin sequence (Figure 8c). Absence of cytoplasmic contamination was confirmed by the lack of a Western blot signal corresponding to UGPase (Supplementary Figure S9b). These results support the hypothesis that nuclear localization of ncRNA3 is dependent on p14 or *p14BA2* binding to the Coremin motif of ncRNA3.

### 3.8. Identification of p14 Proximal Interactors Using TurboID-Based Labeling

**Figure 9:**
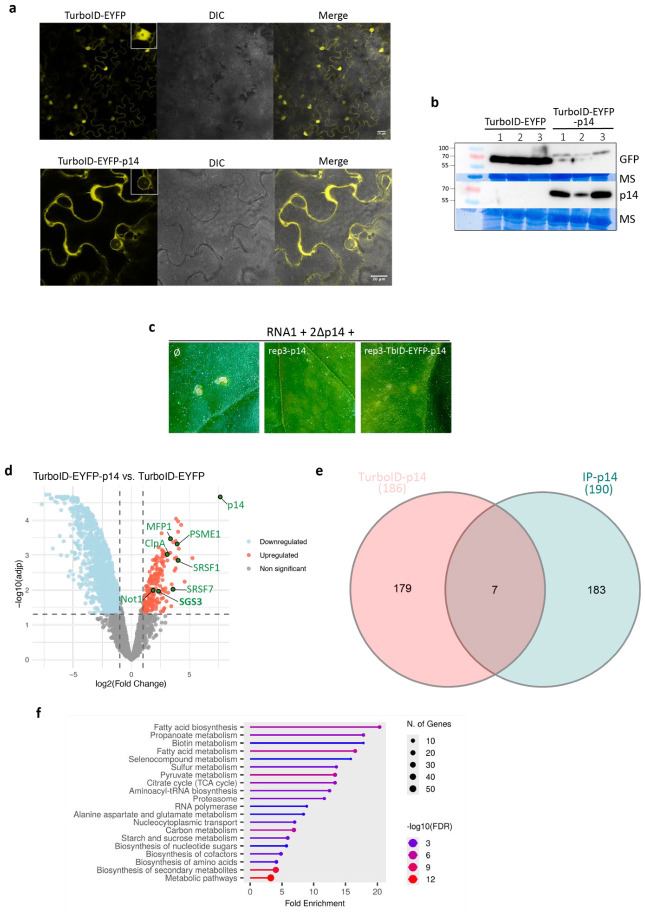
Identification of p14 interacting proteins using proximity labelling coupled to mass spectrometry. (**a**) Subcellular localization of TurboID-EYFP-p14 and TurboID-EYFP. Transiently expressed *N. benthamiana* are observed 40 h post-infiltration on a confocal microscope. A zoomed-in view of a nucleus is shown in the top right angle of the left image to confirm subcellular localization. Scale bars are indicated. (**b**) Western blot analysis of protein expression with GFP and p14 antibodies in the infiltrated leaf samples used for mass spectrometry analysis. MS, membrane staining. (**c**) Complementation assay of TurboID-EYFP-p14. Visualization of the phenotype of local lesions on *C. quinoa*. (**d**) Volcano plot showing proteins identified by TurboID-based proximity labelling in three biologically independent experiments (*n* = 3 for each experiment). Mean log_2_ fold change of three replicates between TurboID-EYFP-p14 and the TurboID-EYFP control was plotted against the -log_10_ adjp. Upregulated proteins (log_2_(FC) > 1, *adjp* < 0.05) are depicted in red while downregulated proteins (log_2_(FC) < −1, *adjp* < 0.05) are depicted in blue, and non-significant proteins (*adjp* > 0.05) are shown in grey. (**e**) Venn diagram showing the overlap between proteins enriched by TurboID-p14 and IP-p14. (**f**) Gene Ontology (GO) term enrichment analysis for specific TurboID-EYFP-p14 enriched proteins.

We extended the analysis of the p14 interactome using TurboID proximity labelling coupled with mass spectrometry. This approach allows the biotinylation of proteins spatially close to TurboID-p14 fusion protein to enable the identification of a broader range of proteins associated with weak, transient or hydrophobic interactions. The functionality and subcellular localization of TurboID-EYFP-p14 were validated through confocal microscopy and complementation assay, respectively. TurboID-EYFP-p14 is predominantly localized in both cytoplasm and nucleolus, consistent with the known localization of p14 [13], while TurboID-EYFP is localized in the cytoplasm and the nucleoplasm (Figure 9a). TurboID-EYFP and TurboID-EYFP-p14 were retrieved at their expected sizes of 63 and 78 kDa, respectively (Figure 9b). TurboID-EYFP-p14 was functional as it complemented a VSR-deficient inoculum (RNA1 + RNA2Δ14) in *C. quinoa* [35], shown by the appearance of chlorotic local lesions following infection with a RNA3-derived vector (rep3) expressing TurboID-EYFP-p14 (Figure 9c). The TurboID-EYFP-p14 construct was transiently expressed in *N. benthamiana* with 200 µM biotin for 6 h, allowing in vivo biotinylation of nearby proteins, regardless of the strength or stability of the interaction. Total proteins were extracted from plant tissues, and free biotin was removed by desalting columns. The successful enrichment of the biotinylated proteins was confirmed by Western blot analyses (Supplemental Figure S10). Following streptavidin enrichment and LC-MS/MS analysis, we identified in total 186 proteins that were significantly enriched (with *padj* < 0.05 and log_2_(fold change) > 1) compared to the TurboID-EYFP control (Figure 9d). We identified an additional set of candidate proteins, including seven previously detected Flag-IP interactors and additional proteins potentially involved in transient or spatially linked processes associated with p14 function (Figure 9e). However, none of the seven proteins shared between the datasets displayed a notable functional relevance. Although DRB4 was detected in the TurboID-EYFP-p14 samples, it was not enriched compared to the control. Notably, several enriched proteins were associated with RNA metabolism and post-transcriptional regulation, including serine/arginine-rich splicing factors (SRSF1 and SRSF7), suggesting a role for p14 in altering host RNA splicing and stability (Figure 9d). Proteins involved in protein degradation including the proteasome activator PSME4 and the ATP-dependent protease ClpA were also identified, suggesting that p14 could modulate protein turnover pathways, possibly to avoid host defence responses. Additionally, the identification of MFP1, a MAR-binding filament-like protein associated with nuclear matrix attachment, further suggests that p14 might interfere with nuclear organization or chromatin structure (Figure 9d). All together, these findings support a model in which p14 alters host RNA processing, protein turnover, and possibly nuclear architecture to support viral infection and suppress antiviral responses (Figure 9f). Interestingly, the SUPPRESSOR OF GENE SILENCING 3 protein (SGS3) was identified among the candidates, which may explain p14′s known role in suppressing RNA interference through the reduction in secondary siRNA accumulation [13]. SGS3 is a cofactor of RNA-dependant RNA polymerase 6 (RDR6), both being essential to produce dsRNA from cleaved RNA fragments generated by primary siRNA-loaded RISC complexes. In the absence of dsRNA production, secondary siRNAs do not accumulate, leading to an inactive RNA silencing transitivity pathway. This finding provides new insight into the mechanism by which p14 suppresses RNA silencing. However, due to time constraints, only interactions between p14 and proteins involved in the transitivity pathway were tested.

### 3.9. Analyses of p14 and RNAi Transitivity Actors

The identification of SGS3 among the in vivo proximity labelled proteins led us to investigate potential interactions between RdR6, SGS3 and the p14 VSR. Y2H assay using RdR6 protein fused to GAL4-AD and GAL4-BD-p14 did not lead to yeast growth on SD-WLH media, suggesting an absence of interaction. We also tested the interaction between the different domains of SGS3 described by Cheng and Wang [36] and GAL4-BD-p14 protein, i.e., SGS3 N-terminal (NTD), Zinc-finger (ZF), rice gene X and SGS3 (XS) domains and Coiled-coiled C-terminal (CC) using full-length and several combinations of domains. A S173D sequence variation was retrieved in all independent NTD and full-length constructions. None of these sequences fused to GAL4-AD were able to interact with GAL4-BD-p14 on SD-WLH media, suggesting an absence of a direct interaction. However, a weak growth was observed for yeasts expressing GAL-BD-p14 together with GAL4-AD-NTD on SD-WLH media.

## 4. Discussion

In this study, we examined the interaction between the BNYVV VSR p14 protein and its hypomorphic *p14BA2* mutant and NbDRB4 using different approaches. Immunoprecipitation identified NbDRB4 as a potential p14 partner, but its interaction was only consistent and reproducible in the case of the hypomorphic mutant *p14BA2* in the absence of ncRNA3 accumulation. We hypothesize that the interaction might be a transient event that has been stabilized by a crosslinking reaction between the cysteine-rich p14 and DRB4, thus facilitating its capture. The absence of enrichment following proximity labelling suggests the absence of a stable direct interaction between *NbDRB4* and the wild-type VSR. The p14 localization was modified upon NbDRB4 co-expression, suggesting that NbDRB4 may induce nucleolar exclusion of p14, a phenomenon possibly linked to a transient interaction between the two proteins or involving a third protein that could bridge the interactions. The p14-NbDRB4 interaction was also observed in yeast two-hybrid experiments, although weaker than the one observed between *p14BA2* and NbDRB4. FRET-FLIM analysis confirmed this interaction, although it was not retrieved in in vitro pull-down assays, which may be due to the lack of essential cofactors or post-translational modifications in bacterial systems, which are present in planta. The virus expressing the *p14BA2* protein is deficient in long-distance movement and is complemented, although partially, by ncRNA3 accumulation. Such a virus is also capable of long-distance movement in RdR6i *N. benthamiana* independently of RNA3 [16]. We investigated the effect of ncRNA3 expression on VSR interactions in vivo and found that ncRNA3 prevented the interaction between *p14BA2* and NbDRB4 without altering their colocalization (Figure 7). Furthermore, *p14BA2*-NbDRB4 interaction was partially disrupted in RDR6 knockdown *N. benthamiana* plants (Figure 4), suggesting that a partial exclusion from the nucleolus and/or loss of NbDRB4 interaction may provide a functional version of the *p14BA2* hypomorphic VSR. If both NbDRB4 and p14 proteins interact with the Coremin motif, only the viral proteins showed interaction specificity. The subcellular fractionation experiments confirmed that the nuclear localization of ncRNA3 is mediated only by p14s able to bind Coremin. Additionally, ncRNA3 disrupted *p14BA2*-NbDRB4 complex *in planta* but this was not observed in yeast, implying the involvement of unidentified host factors mandatory in such a mechanism. As *p14BA2* behaves like p14 in the presence of ncRNA3, we suspect that ncRNA3 might play a role in the refolding of the *p14BA2* protein turning the hypomorphic version into a functional one in *N. benthamiana* while also perturbing NbDRB4 function before dissociating from the complex. This also suggests a potential role of p14 on DRB4 in the transitivity pathway. The function of endogenous DRB4 in *N. benthamiana* is currently poorly described. Unfortunately, the *N.benthamiana* transgenic line expressing NbDRB4-tRFP displays a dwarf and sterile phenotype which prevented performing functional assays.

Interestingly, viral suppressors of RNA silencing (VSRs) such as HC-Pro, p19, and P15 disrupt plant development by interfering with endogenous miRNA and ta-siRNA pathways, leading to misregulation of key developmental regulators like ARF8 and TCP transcription factors [37,38,39]. These disruptions cause floral abnormalities and reduced reproductive performance, often resulting in sterility-like phenotypes, highlighting the essential role of small RNA pathways in fertility. Recent chromosome-level genome assemblies [40,41] show that one copy of DRB4 genes (also true for other RNA silencing-related genes) present in *N. tabacum* has been lost in tetraploid *N. benthamiana*, which likely contributes to its high viral susceptibility. NbDRB4 overexpression may exhibit a silencing suppressor function that perturbs AGO-mediated silencing, causing developmental defects and sterility (Supplementary Figure S7), or may reflect broader effects of its dsRNA-binding activity, illustrating the intricate interplay between viral pathogenicity, small RNA pathways, and reproductive regulation.

While p14 acts on its own, the ncRNA3 nuclear localization may serve a dual purpose. First, it could represent a viral strategy to protect ncRNA3 from cytoplasmic RNA silencing pathway. Sequestration in the nucleus would allow ncRNA3 to avoid cytoplasmic Dicer-like enzyme activity and RDR6-mediated amplification, helping the virus to suppress transitivity-related RNA silencing responses. Second, nuclear localization may position ncRNA3 to interfere with host nuclear processes, such as transcription, RNA maturation, or export, possibly mimicking host RNAs or interacting with nucleolar components recruited by p14 in a manner analogous to other viral ncRNAs that function as molecular decoys. For instance, non-coding RNAs from flaviviruses can suppress antiviral RNA interference by interacting directly with RNAi factors such as Dicer or Argonaute [42]. Together, our findings uncover a new side of regulation in the function of ncRNA3 and suggest that its nuclear localization is tightly controlled by the virus, probably to support its role in host manipulation. Few examples exist for positive-sense RNA viral genome elements entering the nucleus. As an example, the genome of hibiscus chlorotic ringspot virus (HCRSV, a Tombusviridae) enters the nucleus of infected cells through the formation of viral p23 protein-importin-α and viral RNA complex [43]. Conversely, nucleolar localization of viral proteins represents a widespread viral strategy. Nucleoli are involved in various RNA biogenesis, processing and maturation mechanisms involving three major proteins: nucleolin, B23 and fibrillarin [44]. These proteins govern the nucleolus dynamic and RNP (ribonucleoprotein) complex formation and therefore represent a site to reach and operate for viruses. For example, the GRV ORF3 and RSV p2 interact with fibrillarin to ensure viral systemic spread; silencing of fibrillarin was shown to impair the viral systemic movement [45,46,47]. While p14 did not directly interact with NbFibrillarin (NbFib), the VSR could require another bridging partner to recruit this protein and ensure BNYVV long-distance movement through the formation of vRNP [48]; for this purpose, nucleolar targeting of ncRNA3 could be relevant to trigger an unknown mechanism required for viral systemic spread. The p14 silencing suppression function is independent of its nucleolar localization, but it reduces specifically secondary siRNA production ([13] and this study) and limits their movement [16], indicating that p14 acts on the RDR6-dependent transitivity pathway. The use of TurboID proximity labelling enabled the identification of a set of proteins located near the p14 VSR in its native cellular environment. It further allowed the identification of RDR6’s cofactor, SUPPRESSOR OF GENE SILENCING 3 (SGS3), which strengthens our model. RDR6/SGS3 are essential to produce secondary siRNAs that reinforce silencing responses [49]. Several viruses have evolved strategies to directly interact with or degrade SGS3 to suppress host immunity. For example, the VPg protein of Turnip mosaic virus (TuMV) mediates degradation of SGS3 via the ubiquitin-proteasome and autophagy pathways, reducing siRNA accumulation and promoting infection [36]. Similarly, Citrus tristeza virus p20 hijacks the autophagy pathway to degrade SGS3 by forming a ternary complex with ATG8, thereby facilitating viral replication [50]. Additionally, Rice tungro bacilliform virus (RTBV) encodes a viral protein that directly interacts with SGS3 to enhance viral accumulation or act as a virulence factor [51]. Given the established role of SGS3 in antiviral defence and the frequent targeting of this protein by diverse VSRs, its proximity to p14 suggests that p14 may act through a similar strategy—either by sequestration, destabilization, or competitive inhibition of SGS3/RDR6 function. Although a direct interaction between p14 and SGS3 or between p14 and RdR6 was not detected individually by the yeast two-hybrid approach, a possible effect of p14 on the RDR6/SGS3 complex still remains. These results should therefore be considered as preliminary with a possible negative effect of the S173D mutation. This will be further evaluated by yeast two-hybrid bridged assays that could reveal a p14 effect on SGS3 within the RDR6/SGS3 complex. This may also explain the previously reported effect of p14 on reducing secondary siRNAs accumulation [13] and on the transitivity pathway [16]. Interestingly, Clp protease (ClpA), several proteasome subunits and ubiquitin-related proteins were enriched in proximity of p14, including PSME4 and multiple 26S protease regulators. This observation, combined with the enrichment of SGS3, supports the hypothesis that p14, like other VSRs such as TuMV VPg and CTV p20, could promote SGS3 degradation through the host protein degradation machinery. Although ATG8 was not detected, the presence of both ubiquitin-related enzymes and proteasome components suggests that p14 alters protein turnover to avoid degradation of viral proteins or to suppress immune signalling. The enrichment of such proteins involved in protein degradation as well as RNA metabolism and nuclear architecture provides new insights into the potential cellular interactors and functional roles of p14 during viral infection to participate in RNA processing and post-transcriptional regulation. These include two serine/arginine-rich splicing factors, SRSF1 and SRSF7, with both well-documented roles in viral infection. The enrichment of such factors as well as CCR4-NOT (Not1), involved in RNA processing and stability, suggests a possible mechanism where p14 modulates RNA metabolism. A recent study showed that CCR4 is hijacked by barley yellow striate mosaic virus (BYSMV) to enhance viral replication by degrading host mRNAs and releasing viral RNA-binding proteins from inhibitory complexes [52]. Last, identification of MFP1, a MAR-binding nuclear matrix, raises the possibility that p14 may influence nuclear organization or chromatin dynamics in favour of viral replication. While these interactions remain to be validated, our study broadens the functional role of p14. Importantly, the TurboID approach allowed the identification of transient or weak interactions that might have been missed by traditional immunoprecipitation, highlighting the benefits of this methodology for the understanding of dynamic host–pathogen interactions. However, limitations exist in such an approach for interacting proteins missing accessible lysine residues or naturally biotinylated residues that will not be enriched. Immunoprecipitation provided more candidates probably due to indirect interactions for some identified proteins. The seven proteins retrieved by both approaches are Serine/Arginine rich splicing factor 1 (SRSF1), a well-known RNA-binding protein involved in pre-mRNA splicing regulation; Development and cell death (DCD), a protein associated with programmed cell death and stress responses; AGO1B-like involved in RNA silencing; an RNA-binding KH domain-containing protein likely involved in RNA processing; Ribonuclease J, a 5′-3′ exoribonuclease involved in RNA maturation and degradation in bacteria; Elongation factor 3 that plays a role in translation elongation; and the ATP-dependent RNA helicase DHH1 implicated in mRNA turnover and RNA granule dynamics. Altogether, these proteins support a role for p14 in RNA metabolism and gene expression regulation.

Future work involving reverse coIP, RNA immunoprecipitation and nuclear interactome analysis of ncRNA3 will provide further insights into the dynamic interactions and mechanisms underlying p14’s function in viral pathogenesis.

## Data Availability

The mass spectrometry proteomics data have been deposited to the ProteomeXchange Consortium via the PRIDE [53] partner repository with the dataset identifier PXD068024 and 10.6019/PXD068024.

## Supplementary Materials

The following supporting information can be downloaded at: https://www.mdpi.com/article/10.3390/v18070801/s1, Figure S1: Mass spectrometry data from ectopic IP performed on agroinfiltrated Flag-p14 *N. benthamiana*; Figure S2: *N. benthamiana* and *A. thaliana* DRB4 protein sequences alignment; Figure S3: Molecular analyses of proteins and RNA accumulation in *N. benthamiana* leaves expressing eGFP-p14 or eGFP-p14BA2 and NbDRB4-tRFP, in the presence of RNA3 or RNA3E; Figure S4: Colocalization and interaction assay of eGFP-p14 and AtDRB4-tRFP; Figure S5: Protein accumulation in RdR6i *N. benthamiana* leaves expressing eGFP-p14 or eGFP-p14BA2 and NbDRB4-tRFP; Figure S6: Interaction assay between fibrillarin and p14, p14BA2 and NbDRB4 transiently expressed in *N. benthamiana*; Figure S7: Phenotypes of transgenic *N. benthamiana* overexpressing AtDRB4-tRFP and NbDRB4-tRFP fusion proteins; Figure S8: NbDRB4 suppresses GFP silencing in *N. benthamiana* GFP reporter assay; Figure S9: Immunodetection of viral proteins and UGPase and H3 proteins to evaluate fractionation quality; Figure S10: Western blot analysis of biotinylated proteins captured on Streptavidin beads; Table S1: List of primers used in the study.
