# Supplementary material for: A Viral Long Non-Coding RNA Modulates Viral RNA Silencing Suppressor and DCL4-Associated DRB4 Protein Interaction
Source: Viruses. 2026 Jul 20;18(7):801. doi: 10.3390/v18070801 (PMC13431473; doi:10.3390/v18070801)
Supplement: Supplementary file 1 [file viruses-18-00801-s001.zip › viruses-4041160-supplementary.pdf]

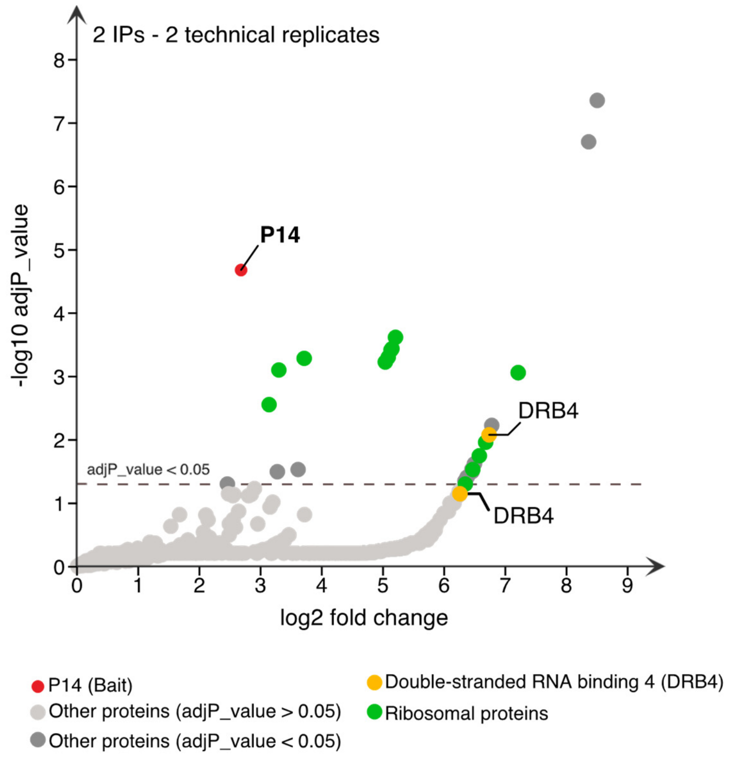

Supplementary Figure S1: Mass spectrometry data from IP performed on agroinfiltrated Flag-p14 *Nicotiana benthamiana*

Volcano plot representation showing the enrichment of proteins co-purified with Flag-p14. Two biological replicates with two technical replicates were performed. The dashed line indicates the threshold above which proteins are significantly enriched (adjusted p-value < 0.05, quasi-likelihood negative binomial generalized log-linear model).

Statistical analysis based on spectral counts was performed through the IPinquiry4 R package (retrieved from https://github.com/hzuber67/IPinquiry4.git). This package calculates fold change and p-values using the quasi-likelihood negative binomial generalized log-linear model implemented in the edgeR package. Common and tagwise dispersions were calculated with the implemented edgeR function by filtering out less abundant proteins that could adversely affect the dispersion estimation. The size factor used to scale samples were calculated according to the DESeq2 normalization method (i.e., median of ratios method). P-value was adjusted using the Benjamini–Hochberg method from stats R package. Dark grey dots with high log2 fc correspond to mitochondrial and chloroplastic proteins - IPR013857 (NADH:ubiquinone oxidoreductase intermediate-associated protein 30) and Plastocyanin A'/A'' IPR001235 (Blue (type 1) copper protein, plastocyanin-type) and were not investigated.

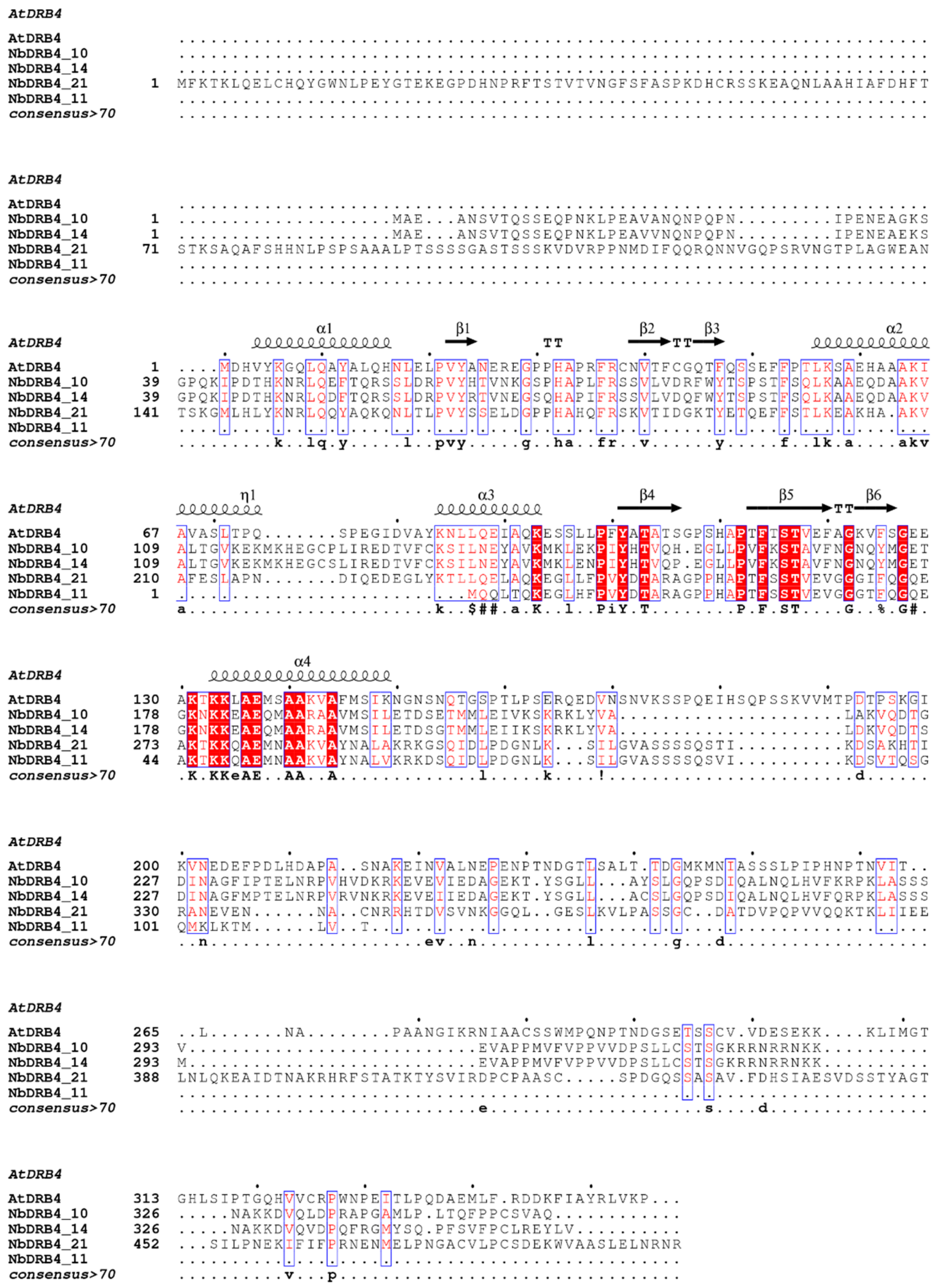

Supplementary Figure S2: *Nicotiana benthamiana* and *Arabidopsis thaliana* DRB4 protein sequences alignment

The four DRB4 protein sequences of *N. benthamiana* were aligned with DRB4 from *A. thaliana* using ESPript3.0 software. AtDRB4 refers to as DRB4 from *A. thaliana*. NbDRB4_10 (Niben101Scf06376g01010), NbDRB4_14 (Niben101Scf09861g01014), NbDRB4_21 (Niben101Scf05841g01021) and NbDRB4_11 (Niben101Scf05841g01011) refer to DRB4 protein from *N. benthamiana* with their accession number indicated here in brackets. The consensus sequence is indicated at the bottom of sequence alignment. Amino-acids conserved at 100 % are highlighted in red and indicated in capital letters in the consensus sequence, whereas those conserved at 70% are written in red and indicated in lowercase letters in the consensus sequence. # represents N (asparagine), D (aspartic acid), Q (glutamine) or E (glutamic acid); $ L (leucine) or M (methionine); % F (phenylalanine) or Y (tyrosine) and ! V (valine) or I (isoleucine) amino acids conservation in the consensus sequence. Alpha helix and beta strands composing DRB4 dsRBD (double-stranded RNA binding domain) from *A. thaliana* are depicted at the top of sequence alignment.

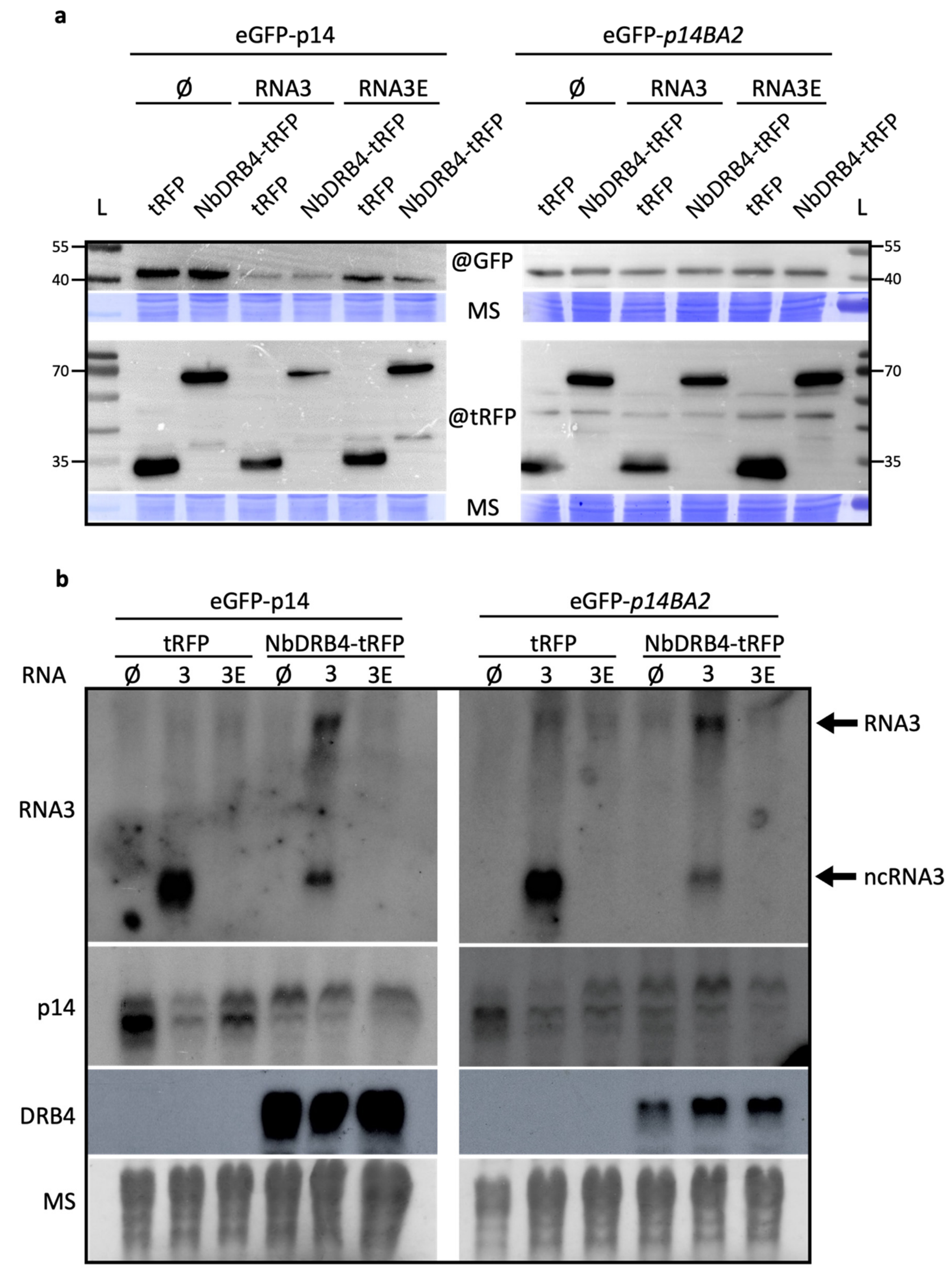

Supplementary Figure S3: Molecular analyses of proteins and RNA accumulation in *Nicotiana benthamiana* leaves expressing eGFP-p14 or eGFP-*p14BA2* and NbDRB4-tRFP, in the presence of RNA3 or RNA3E

Proteins (a) and RNA (b) extracted from *N. benthamiana* transiently expressing eGFP-p14 or eGFP-*p14BA2* and tRFP or NbDRB4-tRFP and RNA3 or RNA3E were analysed by western blot and northern

blot respectively. Antibodies and specific probes used for detection are indicated. RNA3 and ncRNA3 are shown by arrows on the right. Molecular weights (kDa) are indicated in A). MS, membrane staining; L, ladder.

a

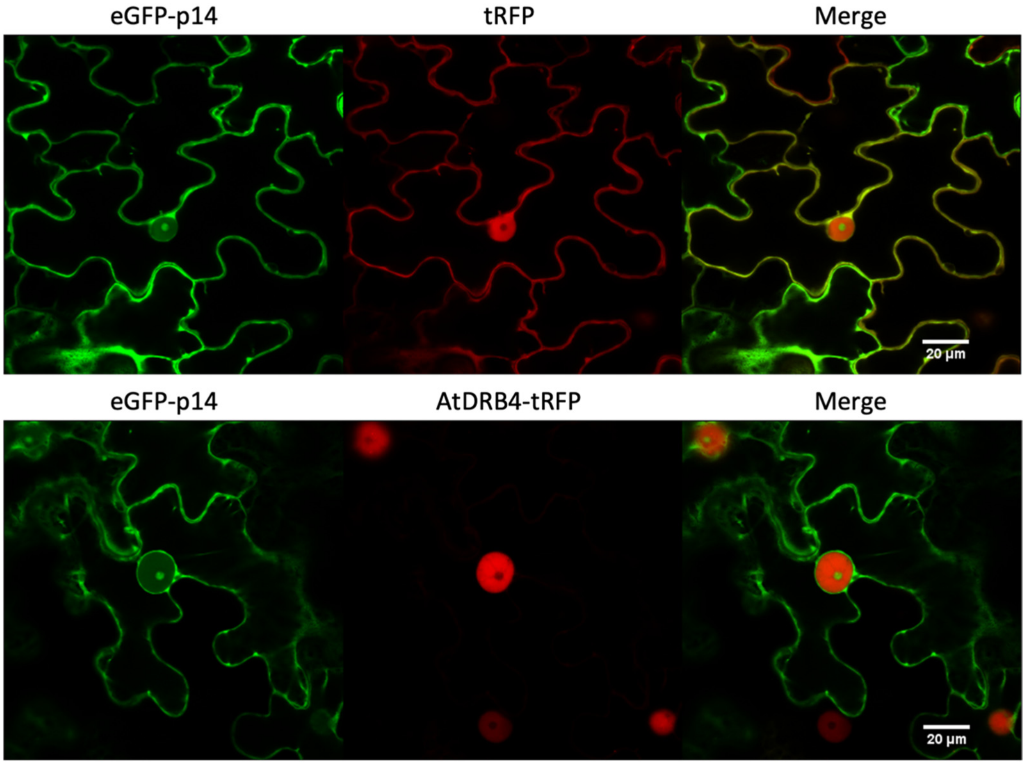

b

	FRET efficiency (%)	
eGFP-p14 + AtDRB4-tRFP	Rep 1	Rep 2
	-2.1%	3.9%

Supplementary Figure S4: Colocalization and interaction assay of eGFP-p14 and AtDRB4-tRFP

a) Transiently expressed eGFP-p14 and tRFP (upper panel) or AtDRB4-tRFP (lower panel). Observations were performed three days post-infiltration on a confocal microscope. Scale bars are indicated. b) FRET efficiency (%) was determined for two independent experiments (Replicate, Rep). No interaction is shown by a yellow background.

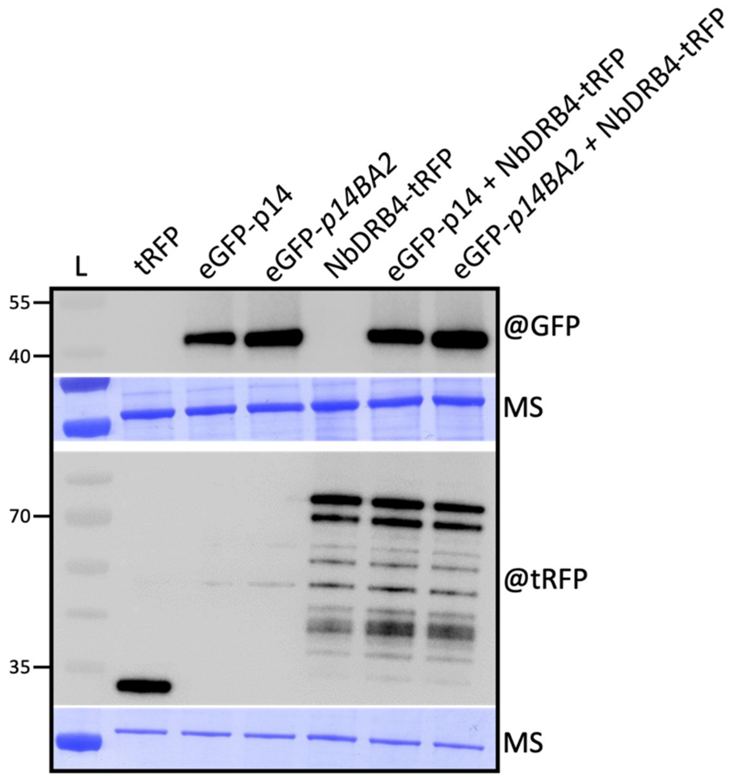

Supplementary Figure S5: Protein accumulation in RDR6i *Nicotiana benthamiana* leaves expressing eGFP-p14 or eGFP-*p14BA2* and NbDRB4-tRFP

Proteins extracted from *N. benthamiana* transiently expressing eGFP-p14 or eGFP-*p14BA2* and tRFP or NbDRB4-tRFP were analysed by western blot. Antibodies used for detection are indicated. Molecular weights (kDa) are indicated. MS, membrane staining; L, ladder.

	FRET efficiency (%)		
NbFib-tRFP +	eGFP-p14	eGFP-*p14BA2*	NbDRB4-eGFP
	-1.2%	-3.1%	-1.6%

Supplementary Figure S6: Interaction assay between fibrillarin and p14, *p14BA2* and NbDRB4 transiently expressed in *Nicotiana benthamiana*

FRET efficiencies (%) were determined in leaves co-expressing NbFib-tRFP and eGFP-p14, eGFP-*p14BA2* or NbDRB4-eGFP. Observations were performed three days post-infiltration. The absence of interaction is shown by a yellow background. For each condition, 20 to 25 nuclei were analysed.

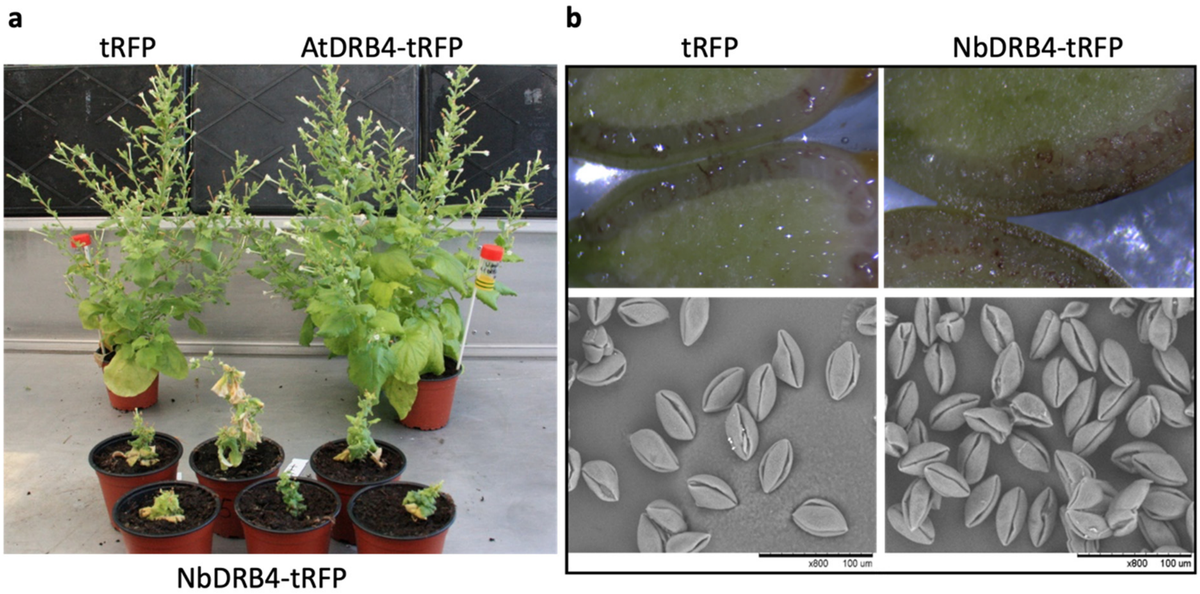

Supplementary Figure S7: Phenotypes of transgenic *Nicotiana benthamiana* overexpressing AtDRB4-tRFP and NbDRB4-tRFP fusion proteins

a) *Nicotiana benthamiana* plants expressing tRFP (upper panel, left), AtDRB4-tRFP (upper panel, right) do not present growth and reproductive defects while those expressing NbDRB4-tRFP (lower panel) are dwarfs and sterile. b) Flowers from transgenic *N. benthamiana* overexpressing tRFP as a control and NbDRB4-tRFP were dissected to observe ovules (upper panel) and pollen (lower panel) with scanning electron microscope.

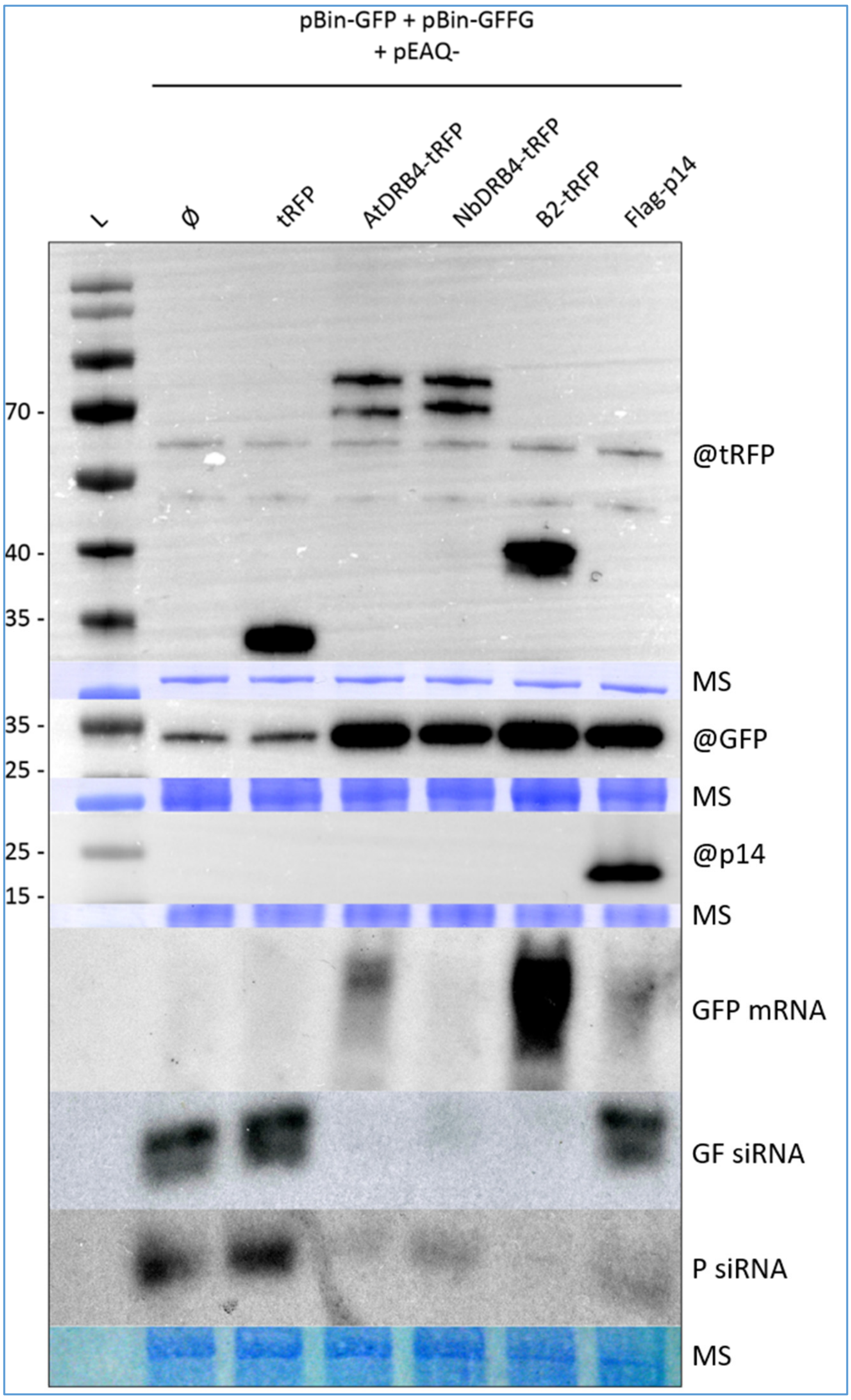

Supplementary Figure S8: NbDRB4 suppresses GFP silencing in *N. benthamiana* GFP reporter assay

Agrobacterium-mediated transient expression of GFP (pBin-GFP), GFFG (pBin-GFFG) and empty vector (pEAQ-Ø) or ensuring the expression of a (potent) silencing suppressor were infiltrated in *N. benthamiana*. Samples were harvested at 3 dpi and analysed for protein accumulation by western blot, GFP messenger RNA by high molecular weight northern blot and siRNA by low molecular weight northern blot. The GFP silencing is monitored by GFP protein, GFP mRNA and GF and P siRNA accumulation levels. Antibodies and probes employed are indicated on the right. Molecular weights (kDa) are indicated on the left. MS, membrane staining; L, ladder.

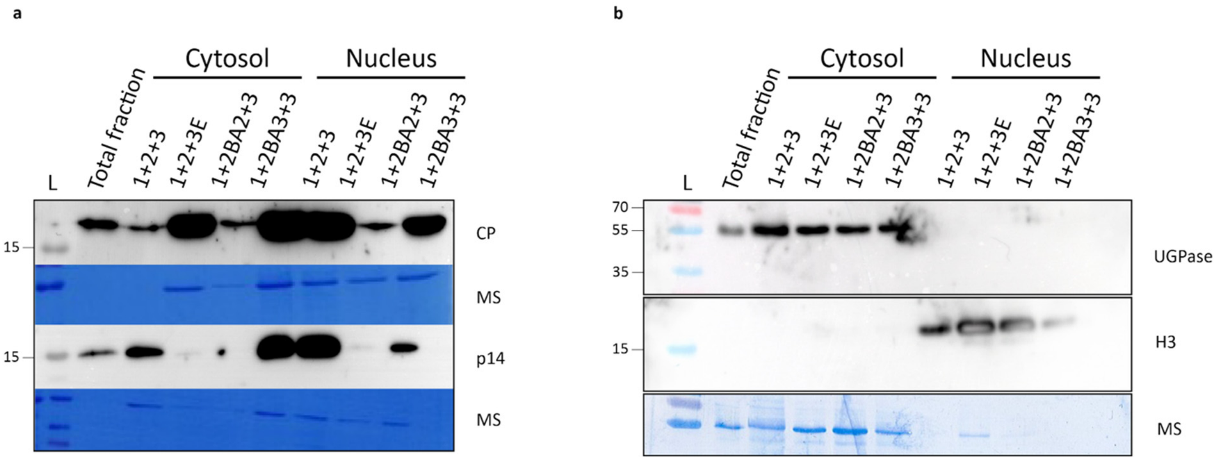

Supplementary Figure S9: Immunodetection of viral proteins and UGPase and H3 proteins to evaluate fractionation quality

Proteins extracted after subcellular fractionation of *N. benthamiana* leaves infected with RNA1, RNA2 wild-type or 2BA2 (expressing *p14BA2*) or 2BA3 (expressing *p14BA3*) and RNA3 or RNA3E were analysed by western blot. Antibodies used for detection are indicated. Molecular weights (kDa) are indicated. MS, membrane staining; L, ladder.

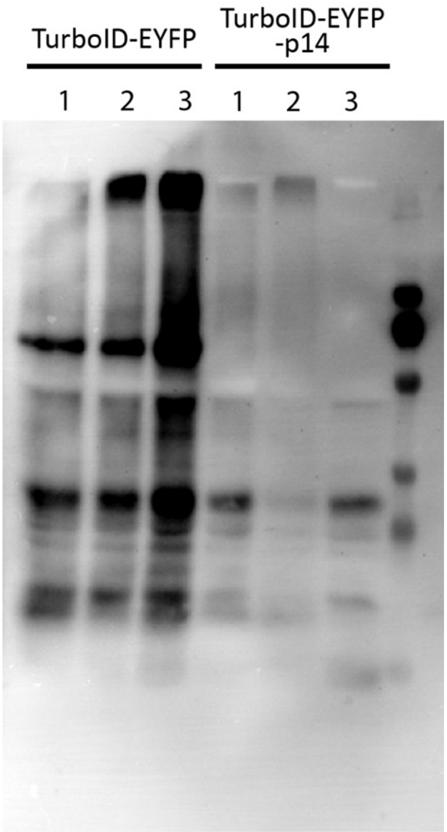

Supplementary Figure S10: Western blot analysis of biotinylated proteins captured on Streptavidin beads

For each construct, there are three independent replicates (1, 2 and 3). Streptavidin-HRP was used for analysis of biotinylated proteins in different samples. TurboID-EYFP is expressed at higher level than TurboID-EYFP-p14, explaining the higher cis-biotinylation in TurboID-EYFP samples (see Fig. 9b)

Supplementary Table S1: List of primers used in the study.

Restriction sites and Gateway Att sequences are highlighted

Primer	Sequence (5' to 3')	Purpose
**ForDRB4**	AAAGAATTCGCAATGGCAGAAGCAAATTCT	Co-localisation and FRET-FLIM
**RevDRB4**	AAAGTCGACATCTCACTGCGCCACCGAGCA	Co-localisation and FRET-FLIM
**ForFib**	AAAGAATTCGCAATGGTTGCACCAACTAGAGGT	Co-localisation and FRET-FLIM
**RevFib**	AAAGTCGACATCCTAGGCAGCAGCCTTCTGCTTCTT	Co-localisation and FRET-FLIM
**For-EcoRI-DRB4**	AAAGAATTCGCA**ATG**GCAGAAGCAAATTCT	Y2H/Y3H
**Rev-SalI-DRB4**	AAAGTCGACATC**TCA**CTGCGCCACCGAGCA	Y2H
**Rev-XmaI-DRB4**	AAACCCGGGATC**TCA**CTGCGCCACCGAGCA	Y3H
**For-His-DRB4**	GCTTAGCTCTTCCAGCGCAGAAGCAAATTCTGTGAC	Pull-down assay
**Rev-DRB4**	GCTTAGCTCTTCCTCACTGCGCCACCGAGCAGGGAGGAAA	Pull-down assay
**For-MBP-p14/BA2**	GCTTAGCTCTTCCAGCGGGATGGTAGATAGTTTGTG	Pull-down assay
**Rev-p14/BA2**	GCTTAGCTCTTCCTCACACCTCAGGATCGACAATAACATG	Pull-down assay
**pGAD-RDR6-FW**	atcttgttgacaatatatgaCCCGGGTGGGCATCGATAC	Gibson-Y2H
**pGAD-RDR6-REV**	tcagagccctctgatcccatGAATTCACTGGCCTCCATGG	Gibson-Y2H
**RDR6-pGADFW**	ccatggaggccagtgaattcATGGGATCAGAGGGCTCTG	Gibson-Y2H

**RDR6-pGADREV**	cgtatcgatgcccacccgggTCATATATTGTCAACAAGATACCTTC	Gibson-Y2H
**SGS3-gwF**	GGGGACAAGTTTGTACAAAAAAGCAGGCTTCATGAGTTCAAGCAAAGGGGTTG	Entry clone production
**SGS3-gwR**	GGGGACCACTTTGTACAAGAAAGCTGGGTCCTATTGAGATTGCTCTGGGGAGTACTT	Entry clone production
**SGS3NTD-gwR**	GGGGACAAGTTTGTACAAAAAAGCAGGCTTCATGAGTTCAAGCAAAGGGGTTG	Entry clone production
**SGS3-ZFgwF**	GGGGACAAGTTTGTACAAAAAAGCAGGCTTCAAAAATCGTTGGATCAAAG	Entry clone production
**SGS3-ZFgwR**	GGGGACCACTTTGTACAAGAAAGCTGGGTCCTACCATCTTCCATACACTTCACC	Entry clone production
**SGS3-XSgwF**	GGGGACAAGTTTGTACAAAAAAGCAGGCTTCGATGGCATAGAGTTTAAAG	Entry clone production
**SGS3-XSgwR**	GGGGACCACTTTGTACAAGAAAGCTGGGTCCTATATGTCTCTTTTCTCTGCCATG	Entry clone production
**SGS3-CCgwF**	GGGGACAAGTTTGTACAAAAAAGCAGGCTTCGACAGCTTTAACCAGCATTC	Entry clone production
